# Interference between ER stress-related bZIP-type and jasmonate-inducible bHLH-type transcription factors in the regulation of triterpene saponin biosynthesis in *Medicago truncatula*

**DOI:** 10.3389/fpls.2022.903793

**Published:** 2022-09-30

**Authors:** Bianca Ribeiro, Marie-Laure Erffelinck, Elia Lacchini, Evi Ceulemans, Maite Colinas, Clara Williams, Evelien Van Hamme, Rebecca De Clercq, Maria Perassolo, Alain Goossens

**Affiliations:** ^1^ Department of Plant Biotechnology and Bioinformatics, Ghent University, Ghent, Belgium; ^2^ VIB Center for Plant Systems Biology, Ghent, Belgium; ^3^ VIB Bio Imaging Core, Ghent, Belgium; ^4^ Cátedra de Biotecnología, Departamento de Microbiología, Inmunología y Biotecnología, Facultad de Farmacia y Bioquímica, Universidad de Buenos Aires, Buenos Aires, Argentina; ^5^ Instituto de Nanobiotecnología (NANOBIOTEC), Consejo Nacional de Investigaciones Científicas y Técnicas-Universidad de Buenos Aires, Buenos Aires, Argentina

**Keywords:** triterpene, saponin, *Medicago catharanthus*, *catharanthus*, jasmonate, endoplasmic reticulum, bZIP, basic helix-loop-helix

## Abstract

Triterpene saponins (TS) are a structurally diverse group of metabolites that are widely distributed in plants. They primarily serve as defense compounds and their production is often triggered by biotic stresses through signaling cascades that are modulated by phytohormones such as the jasmonates (JA). Two JA-modulated basic helix-loop-helix (bHLH) transcription factors (TFs), triterpene saponin biosynthesis activating regulator 1 (TSAR1) and TSAR2, have previously been identified as direct activators of TS biosynthesis in the model legume *Medicago truncatula*. Here, we report on the involvement of the core endoplasmic reticulum (ER) stress-related basic leucine zipper (bZIP) TFs bZIP17 and bZIP60 in the regulation of TS biosynthesis. Expression and processing of *M. truncatula* bZIP17 and bZIP60 proteins were altered in roots with perturbed TS biosynthesis or treated with JA. Accordingly, such roots displayed an altered ER network structure. *M. truncatula* bZIP17 and bZIP60 proteins were shown to localize in the nucleus and appeared to be capable of interfering with the TSAR-mediated transactivation of TS biosynthesis genes. Furthermore, interference between ER stress-related bZIP and JA-modulated bHLH TFs in the regulation of JA-dependent terpene biosynthetic pathways may be widespread in the plant kingdom, as we demonstrate that it also occurs in the regulation of monoterpene indole alkaloid biosynthesis in the medicinal plant *Catharanthus roseus*.

## Introduction

Plants are continuously challenged with biotic and abiotic stresses. To cope specifically with biotic stresses, such as herbivore feeding or pathogen attack, plants can trigger the biosynthesis of various classes of specialized defense metabolites. A well-known class is that of the structurally and functionally diverse triterpene saponins (TS), which are produced in distinct plant species, including legumes such as *Medicago truncatula*, and which are particularly valuable for pharmaceutical and agrochemical purposes (Chang and Keasling, 2006; Ajikumar et al., 2008; Kumari et al., 2013; Moses et al., 2014a; Netala et al., 2015). Phytohormones play an essential role in the stress-induced elicitation of these compounds, again illustrated by the fact that *M. truncatula* TS production is transcriptionally controlled by a JA signaling cascade (Suzuki et al., 2002; De Geyter et al., 2012; Pollier et al., 2013a; Mertens et al., 2016a; Goossens et al., 2017).

The early committed steps in TS biosynthesis occur mainly at the endoplasmic reticulum (ER) membrane and start from 2,3-oxidosqualene, which is the last common precursor with the phytosterols. Subsequent cyclization of 2,3-oxidosqualene by saponin-specific 2,3-oxidosqualene cyclases (OSC)s, more specifically β-amyrin synthase (BAS) in *M. truncatula*, yields the pentacyclic oleanane-type triterpene backbone β-amyrin (**Supplementary Figure 1**). Subsequent competitive action of two cytochrome P450-dependent monooxygenases (P450s) results in branching of the *M. truncatula* TS biosynthetic pathway, resulting in the production of two specific classes: the haemolytic and non-haemolytic TS (Gholami et al., 2014). The haemolytic TS branch is defined by three consecutive oxidations at position C-28 of β-amyrin by the P450 CYP716A12, thereby yielding oleanolic acid (Carelli et al., 2011; Fukushima et al., 2011). Positions C-2 and C-23 can be further oxidized by respectively CYP72A67 and CYP72A68v2 (Fukushima et al., 2013; Biazzi et al., 2015). The non-haemolytic branch starts with an oxidation reaction at position C-24 of β-amyrin, catalyzed by CYP93E2, and thereby precluding oxidation at position C-28 (Fukushima et al., 2013; Moses et al., 2014b). Subsequent oxidation at position C-22 by CYP72A61v2 yields soyasapogenol B (Fukushima et al., 2013). UDP-dependent glycosyltransferases (UGTs) can further decorate the triterpene aglycones through attachment of sugar moieties, additionally diversifying the TS compendium (Seki et al., 2015) (**Supplementary Figure 1**).

The past decades, great progress has been made in the quest for transcription factors (TFs) that are modulated by JA or other cues and that control the production of specialized metabolites in plants (De Geyter et al., 2012; Zhou and Memelink, 2016; Goossens et al., 2017; Colinas and Goossens, 2018; Shoji, 2019). Particularly relevant are the basic helix-loop-helix (bHLH) TFs (Goossens et al., 2017). MYC2 was the first bHLH TF reported to control different branches of terpene biosynthesis in *Arabidopsis thaliana*, *Solanum lycopersicum* and *Artemisia annua*, among others (Hong et al., 2012; Kazan and Manners, 2013; Spyropoulou et al., 2014; Goossens et al., 2017). Later, in the medicinal plant *Catharanthus roseus*, source of the anti-cancer drugs vinblastine and vincristine, both MYC2 as well as MYC2-unrelated bHLH TFs, such as bHLH iridoid synthesis 1 (BIS1) and BIS2, were found to elicit the monoterpenoid branch of the monoterpenoid indole alkaloid (MIA) pathway (Zhang et al., 2011; Van Moerkercke et al., 2015; Van Moerkercke et al., 2016; Goossens et al., 2017; Schweizer et al., 2018; Liu et al., 2021). Likewise, the *M. truncatula* orthologs of the BIS TFs, i.e. triterpene saponin biosynthesis activating regulator 1 (TSAR1) and TSAR2, were reported to transcriptionally regulate the non-haemolytic and haemolytic branch of TS biosynthesis, respectively (Mertens et al., 2016a).

Posttranslational regulatory mechanisms of TS biosynthesis have also been described (Hemmerlin, 2013; Erffelinck and Goossens, 2018). Particularly, the JA-inducible really interesting new gene (RING) membrane-anchor (RMA) E3 ubiquitin ligase makibishi 1 (MKB1) has been reported to control TS biosynthesis in *M. truncatula* by targeting 3-hydroxy-3-methylglutaryl-CoA reductase (HMGR), a rate-limiting enzyme in sterol and TS precursor biosynthesis, for degradation by the 26S proteasome (Pollier et al., 2013a). MKB1 forms part of the ER-associated degradation (ERAD) machinery, which monitors the correct folding of membrane and secretory proteins whose biogenesis takes place in the ER. Recently, we also identified a heat shock protein 40 that interacts with MKB1 to support its activity (Erffelinck et al., 2021).When plants are evoked with environmental stresses, a programed defense response is launched, in which the ERAD, the unfolded protein response (UPR) and other ER stress responses play an important role (Malhotra and Kaufman, 2007; Liu and Howell, 2010b). Eukaryotic cells have developed signaling networks in response to ER stress, through ER stress sensors that are tethered at the ER membrane. In the model plant *A. thaliana*, several ER stress-specific sensors, including the RNase inositol-requiring enzyme 1 (IRE1), the basic leucine zipper (bZIP) TFs bZIP17 and bZIP28, and the NAC TFs NAC062 and NAC089, have been reported (Liu et al., 2007a; Iwata et al., 2008; Liu and Howell, 2010a; Moreno et al., 2012; Yang et al., 2014a; Yang et al., 2014b; Henriquez-Valencia et al., 2015; Kim et al., 2018; Howell, 2021). The primary target of IRE1 in response to ER stress is *bZIP60* mRNA, which is spliced, causing a frame shift and thereby the elimination of the transmembrane domain of the bZIP60 TF at translation. This truncated version is consequently translocated to the nucleus, where it can install a specific ER stress response. An analogous ER-to-nucleus translocation occurs with bZIP17 and bZIP28, but through proteolytic cleavage (Liu et al., 2007a; Liu et al., 2007b; Howell, 2021).

Here, we explored the regulatory interplay between JA and ER stress signaling in the model legume *M. truncatula*. We demonstrate that *M. truncatula* bZIP17 and bZIP60 can interfere with the transactivation of TS-specific gene promoters by the JA-responsive TSAR1 and TSAR2 TFs and thereby modulate the output of the JA response. We also provide evidence that the interplay between these two TF sets may be conserved in the plant kingdom by demonstrating that it also occurs in the regulation of terpene biosynthesis in the distinct plant *C. roseus*.

## Materials and methods

### DNA constructs

Sequences of the full-length ORFs of *bZIP17* (*Medtr7g088890*) and *bZIP60* (*Medtr1g050502*) were obtained from the *M. truncatula* genome version 4.0 (Tang et al., 2014) and were cloned using Gateway^®^ technology (Invitrogen). Full-length and spliced coding sequences for *bZIP17* and *bZIP60* were PCR amplified (for primers, see **Supplementary Table 1**) and recombined into the donor vector pDONR221. After sequence verification, the entry clones were recombined with the destination vector p2GW7 for *Nicotiana tabacum* protoplast assays (Vanden Bossche et al., 2013). The promoter regions of *HMGR1*, *CYP93E2*, *HMGR4*, *CYP72A67, UGT73F3* and *BAS* recombined with the vector pGWL7 and the full-length coding sequences for *TSAR1* and *TSAR2* recombined with the destination vector p2GW7 had previously been obtained (Mertens et al., 2016a). For the generation of *M. truncatula* hairy roots, sequence-verified entry clones were recombined with the destination vector pK7WG2D for overexpression and pK7GWIWG2(II) for silencing (Karimi et al., 2007). Primers used for cloning of overexpression and silencing constructs and for quantitative reverse transcription PCR (qRT-PCR) analysis are reported in **Supplementary Table 1**.

The coding sequences of *C. roseus* bZIP17 and bZIP60 were amplified from *C. roseus* var. “Little bright eyes” cDNA with Q5^®^ High-Fidelity DNA Polymerase (New England BioLabs^®^) and recombined into the entry vector pDONR221 (Gateway^®^). After sequence verification, the entry clones were recombined with the destination vector p2GW7 (Karimi et al., 2002) for *N. tabacum* protoplast assays (Vanden Bossche et al., 2013). The promoter regions of *GES, G10H* and *IS* recombined with the vector pGWL7 (Karimi et al., 2002) and the full-length coding sequence for *BIS1* recombined with the destination vector p2GW7 (Karimi et al., 2002) had previously been obtained by Van Moerkercke et al. (2015). For expression in flower petals under control of the *CaMV35S* promoter, entry clones were recombined with pK7WG2D (Karimi et al., 2002), using LR Clonase™ enzyme mix (ThermoFisher).

Flower petals of *C. roseus* var. “Little bright eyes” plants (grown under greenhouse conditions) were infiltrated with *Agrobacterium tumefaciens* C58C1 harboring the constructs for overexpression as previously described (Schweizer et al., 2018).

### Generation and cultivation of *Medicago truncatula* hairy roots

Sterilization of *M. truncatula* seeds (ecotype Jemalong J5), transformation of seedlings by *A. rhizogenes* (strain LBA 9402/12), and the subsequent generation of hairy roots were carried out as described previously (Pollier et al., 2011). Hairy roots were cultivated for 21 d in liquid medium to provide proper amounts for RNA extraction.

For the elicitation of TS pathway gene expression, 100 µM methyl jasmonate (MeJA) was added to the medium. For the induction of ER stress, 300 mM NaCl, 0.5 mM SA, or 2 mM DTT was added to the medium. Given that we anticipated that the MeJA concentration that we typically employ to elicit TS biosynthesis may be too strong and possibly mimic antagonistic effects of the ER stress agents, we first determined the minimal MeJA concentration with which TS pathway gene expression could still be induced. qRT-PCR analysis of control (CTR) *M. truncatula* hairy root lines indicated that at a concentration of 5 µM MeJA, a pronounced and significant induction of TS pathway gene expression could still be observed (**Supplementary Figure 2**). This concentration was used for all further experimentation, except for SA, which, as a reported potent JA antagonist of the JA signaling pathway in *A. thaliana* (Van Der Does et al., 2013), was still combined with a MeJA concentration of 100 µM.

### Confocal microscopy

Control and MKB1^KD^ (knock-down line of *MKB1*) (Pollier et al., 2013a) hairy roots were cultivated in nutritive liquid medium (Murashige and Skoog with vitamins supplemented with 1% sucrose) for 2 w and treated with 100 µM MeJA or ethanol (mock treatment) for 24 h. Confocal images (16-bit) were captured with an LSM880 confocal microscope equipped with an Airyscan detector (Zeiss, Jena, Germany). Images were taken in super-resolution, FAST mode by using a Plan-Apochromat 63x/1.4 oil objective (1584 × 1584, pixel size: 43 nm × 43 nm). EGFP was excited using the 488-nm line of an Argon laser (30%) and emission was captured between 495 and 550 nm. Z-sections were made every 185 nm. Images were calculated through pixel reassignment and Wiener filtering by using the built-in “Airyscan Processing” command in the Zen software.

Subcellular localization of the bZIP proteins was determined *via Agrobacterium*-mediated transient expression in *N. benthamiana* epidermal cells. bZIP proteins, either full length or truncated, were fused at the C-terminus of eGFP using the Gateway^®^ vector pB7WGF2. To verify subcellular localization, target proteins were co-transformed together with fluorescent markers for nucleus and ER using pB7m34GW:p35S::NLS-3XCERULEAN and pH7WG2::KDEL-RFP637, respectively. Each vector used was transformed in the *A. tumefaciens* strain C58C1 and a bacterial absorbance A600 nm of 0.8 was used for infiltrating each construct. 72 h after infiltration, leaf sections were collected and fluorescence analyzed by confocal microscopy using a Zeiss LSM710 laser scanner microscope with Plan-Apochromat 20x/0.8 M27. The nuclear marker CFP was excited using a 405-nm laser, while 518-nm and 488-nm Argon lasers were adopted for excitation of the ER RFP marker and GFP::bZIPs fusions, respectively. Z-sections were taken every 2 um. Hairy roots and *N. benthamiana* infiltration images were processed generating maximum intensity projections and adding scale bars using Fiji software.

### Phylogenetic analysis

The bZIP proteins from *A. thaliana* and *M. truncatula* were selected based on Dröge-Laser et al. (2018) and Wang et al. (2015), respectively. The amino acid sequences of all selected bZIP proteins were obtained through PLAZA (Van Bel et al., 2018) and the *Catharanthus roseus* Functional Genomics Database (croFGD; http://bioinformatics.cau.edu.cn/croFGD/) (She et al., 2019). These were aligned using MAFFT. The conserved blocks were determined using GBlocks 0.91b and manual curation. IQTREE was used for model selection (Kalyaanamoorthy et al., 2017), after which the best substitution model was selected; the maximum likelihood phylogenetic tree was generated using 1000 bootstrap replicates (Nguyen et al., 2015). The tree figure was made using FigTree software.

### RNA-Seq analysis

Total RNA of three independent transformant lines per construct was submitted to VIB Nucleomics Core (VIB, Leuven) for Illumina NextSeq500 RNA sequencing (75 nt, single-end read). As described (Pollier et al., 2013b) and using default parameters, the raw RNA-Seq reads were quality-trimmed and mapped on the *M. truncatula* genome v4.0 (Tang et al., 2014) with TOPHAT v2.0.6. Uniquely mapped reads were counted and FPKM values were determined with CUFFLINKS version v2.2.1 (Trapnell, 2013). Differential expression analyses were performed using Cuffdiff (Trapnell, 2013). RNA-Seq data have been deposited in the ArrayExpress database (accession E-MTAB-11668).

### Semi-quantitative qRT-PCR analysis

Frozen hairy roots were ground and the material was used to prepare total RNA and first-strand complementary DNA using the RNeasy Mini Kit (Qiagen) and the iScript cDNA Synthesis Kit (Bio-Rad), respectively, according to each manufacturer’s instructions. qRT-PCR primers for *bZIP17* and *bZIP60* were designed using Beacon Designer 4 (Premier Biosoft International) (**Supplementary Table 1**). The *M. truncatula 40S ribosomal protein S8* and *translation elongation factor 1a* were used as reference genes. The qRT-PCRs were carried out with a LightCycler 480 (Roche) and the LightCycler 480 SYBR Green I Master Kit (Roche) according to the manufacturer’s guidelines. Three replicates were made for each reaction and the relative expression levels using multiple reference genes were calculated using qBase (Hellemans et al., 2007).

### LC-MS and data analysis

*M. truncatula* hairy root samples were extracted as described (Pollier et al., 2011; Ribeiro et al., 2020) and subjected to Ultra Performance Liquid Chromatography High Resolution Mass Spectrometry (UPLC-HRMS) at the VIB Metabolomics Core Ghent (VIB-MCG). 10 µl was injected on a Waters Acquity UHPLC device connected to a Vion HDMS Q-TOF mass spectrometer (Waters, Manchester, UK). Chromatographic separation was carried out on an ACQUITY UPLC BEH C18 (150 × 2.1 mm, 1.7 μm) column (Waters, USA); column temperature was maintained at 40°C. A gradient of two buffers was used for separation: buffer A (99:1:0.1 water:acetonitrile:formic acid, pH 3) and buffer B (99:1:0.1 acetonitrile:water:formic acid, pH 3), as follows: 99% A buffer decreased to 50% A from 0 to 30 min, decreased to 30% from 30 to 35 min, and decreased to 0% from 0 to 37 min. The flow rate was set to 0.35 mL min−1. Electrospray ionization (ESI) was applied, LockSpray ion source was operated in negative ionization mode under the following specific conditions: capillary voltage, 2.5 kV; reference capillary voltage, 3 kV; source temperature, 120°C; desolvation gas temperature, 550°C; desolvation gas flow, 800 L h−1; and cone gas flow, 50 L h−1. The collision energy for full MS scan was set at 6 eV for low energy settings, for high energy settings (HDMSe) it was ramped from 20 to 70 eV. Mass range was set from 120 to 2000 Da, scan time was set at 0.1 s. Nitrogen (greater than 99.5%) was employed as desolvation and cone gas. Leucine-enkephalin (100 pg μL−1 solubilized in water:acetonitrile 1:1 [v/v], with 0.1% formic acid) was used for the lock mass calibration, with scanning every 2 min at a scan time of 0.1 s. Profile data was recorded through Unifi Workstation v2.0 (Waters). Data processing was done with Progenesis QI v2.4 (Waters).

Data were pre-processed by removing features with constant or infinity values across samples. Zero values were replaced by 1.0e-10 for statistical processing. Subsequently, data were transformed using the Arch-sinh function and scaled using the Pareto method. Each condition was analyzed in triplicate on three independent biological replicates for both CTR and bZIP17^KD^ lines. Data were analyzed by two-way ANOVA considering only features with a p value ≤ 0.01 across samples. Ontology for selected candidate TS was determined by comparison with in-house metabolites databases integrated with ChEBI features (Chemical Entities of Biological Interest - EMBL-EBI) as well as by matching features against previously identified TS (Pollier et al., 2011; Ribeiro et al., 2020).

### Transient expression assays in protoplasts

Transient expression assays in *N. tabacum* BY-2 protoplasts were carried out as described by Vanden Bossche et al. (2013). Protoplasts were transfected with a reporter, an effector, and a normalizer plasmid. The effector plasmids contained the *TSAR*, *BIS*, or *bZIP* ORFs driven by the *CaMV35S* promoter; the reporter plasmids contained the *FIREFLY LUCIFERASE* (*fLUC*) ORF under control of the target promoters. The normalization plasmid contained the *Renilla luciferase* (*rLUC*) under control of the *CaMV35S* promoter. Protoplasts were incubated overnight and lysed. fLUC and rLUC readouts were collected using the Dual-Luciferase^®^ Reporter Assay System (Promega). Each assay incorporated eight biological repeats. Promoter activities were normalized by dividing the fLUC values with the corresponding rLUC values and the average of the normalized fLUC values was calculated and set out relatively to the control fLUC values, i.e. measured in protoplasts transfected with an effector plasmid carrying a *GUS* control gene.

## Results

### Confocal imaging exposes an altered ER network structure in *Medicago truncatula* MKB1^KD^ hairy roots

**Figure 1:**
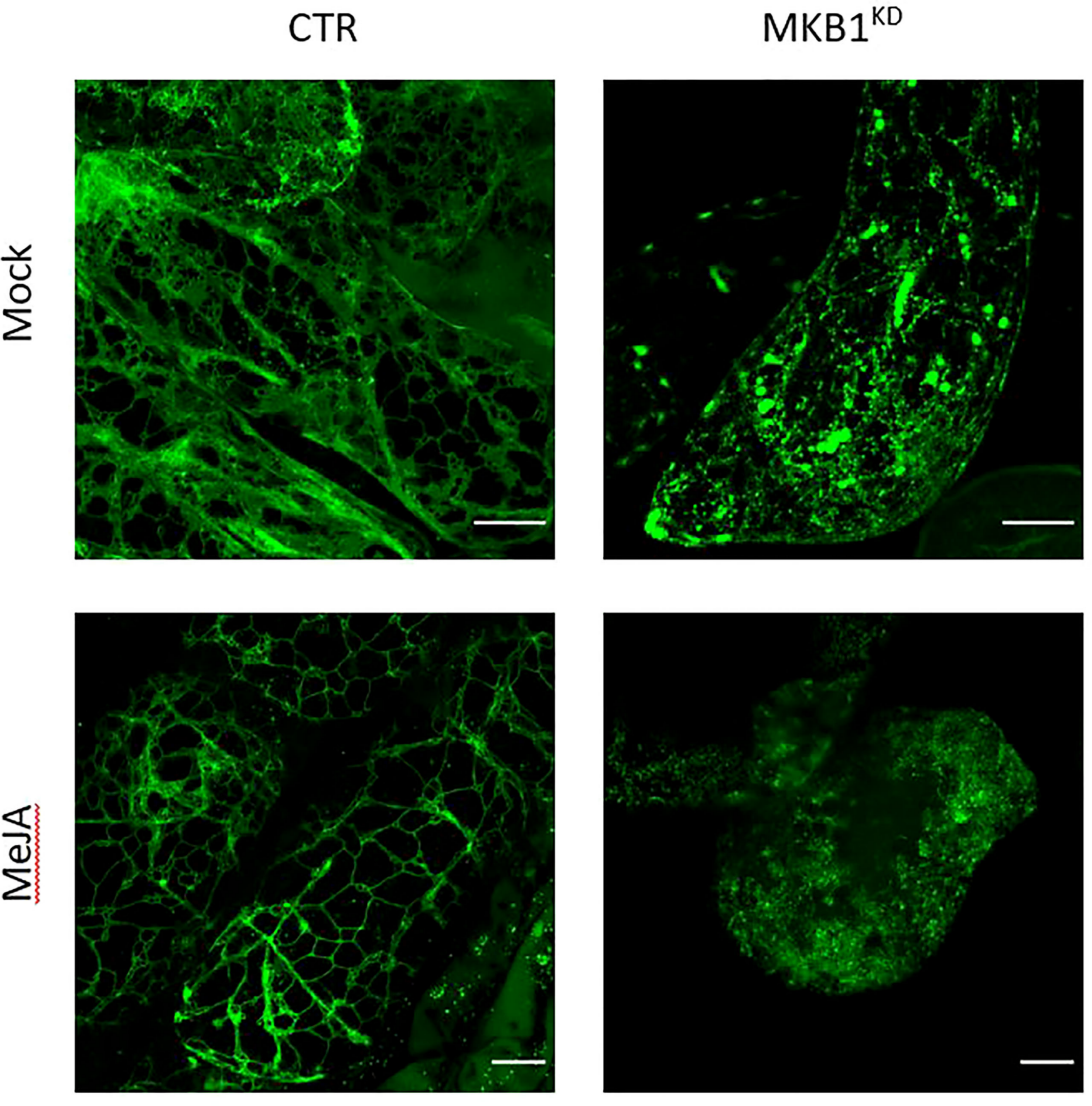
Silencing of *MKB1* and MeJA elicitation alters the ER network structure. Shown are maximum intensity projections of images obtained by Airyscan microscopy of ER-targeted GFP in stably transformed CTR and MKB1^KD^ *M. truncatula* hairy roots. Left, CTR roots elicited (24 h) with ethanol (mock) or 100 µM MeJA. Right, mock- and MeJA-treated MKB1^KD^ roots. Scale bars = 20 μm.

Previously, we have shown that silencing of *MKB1* in *M. truncatula* hairy roots (MKB1^KD^) results in dissociated roots with caltrop-like structures and a perturbed TS profile, accompanied by a TS-specific negative transcriptional feedback (Pollier et al., 2013a). We hypothesized that perturbed ER functionality could trigger an ER-inherent mechanism to manage ER capacity and integrity, and thereby (in)directly modulate TS metabolism. Therefore, we performed confocal imaging to monitor the ER network structure of *M. truncatula* MKB1^KD^ and CTR hairy roots. Hereby, we exploited the fact that the MKB1^KD^ hairy roots also ectopically express ER-targeted GFP (GFP-KDEL) under the control of a *rolD* promoter, which is used as a visual marker for transformation. The ER network structure was notably altered in MKB1^KD^ hairy roots when compared to CTR lines, for instance exhibiting less of the characteristic three-way junctions (**Figure 1**). These phenotypical features were even more pronounced in MKB1^KD^ hairy roots that were elicited with MeJA compared to mock-treated MKB1^KD^ hairy roots. Interestingly, MeJA treatment of CTR roots also led to a visual alteration of the ER network structure, but the effect was distinct from or far less pronounced than the effect caused by loss of MKB1 function (**Figure 1**).

### ER stress marker genes are transcriptionally upregulated in MKB1^KD^ hairy roots

**Figure 2:**
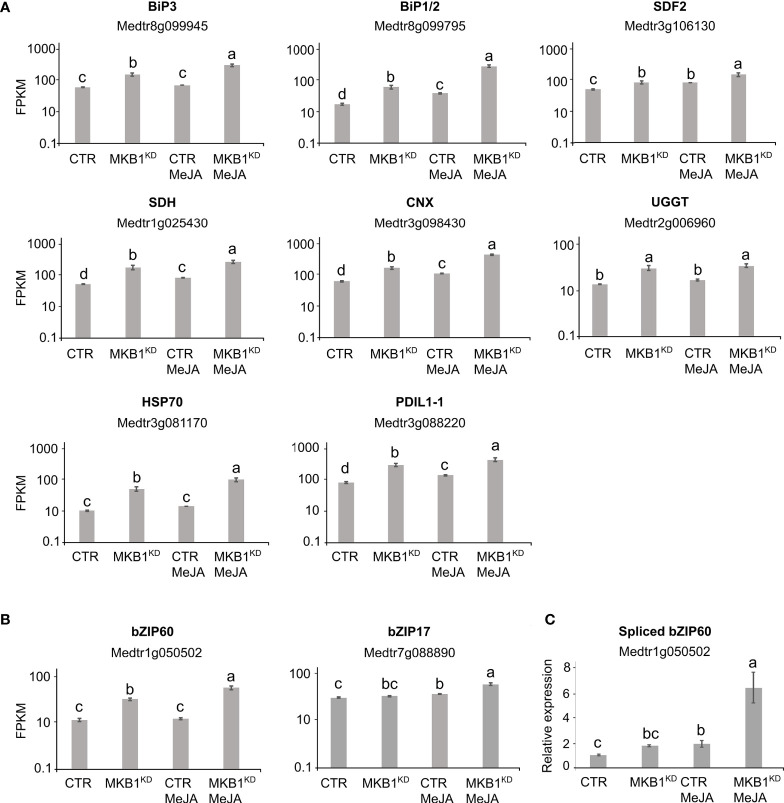
Silencing of *MKB1* and MeJA elicitation both trigger an ER stress response in *M. truncatula*. **(A, B)** RNA-Seq analysis of *M. truncatula* orthologs of *A. thaliana* ER stress marker **(A)** and bZIP TF **(B)** genes in CTR and MKB1^KD^ roots, mock- or MeJA-treated for 4 h. The Y-axis represents the normalized fragments per kb of exon per million fragments mapped (FPKM) values. Error bars designate SE (n = 3, except for MeJA-elicited CTR where n = 2). **(C)** qRT-PCR analysis for the detection of spliced *bZIP60* transcripts in CTR and MKB1^KD^ roots, mock- or MeJA-treated for 4 h. The error bars designate SE (n = 3). Different letters indicate statistically significant differences at P < 0.05 as determined by ANOVA, *post hoc* Tukey analysis.

Given that MKB1^KD^ hairy roots display an altered morphology with irregular cell shapes and the absence of intercellular spaces (Pollier et al., 2013a), the differences in ER network structure may not necessarily reflect ER stress caused by the perturbed ERAD machinery but rather an intracellular reorganization following the modifications in the cellular structure. Therefore, a transcript profiling study by RNA-sequencing (RNA-Seq) was performed on three independent *M. truncatula* CTR and MKB1^KD^ hairy root lines, either mock- or MeJA-treated. A total of 415,338,234 single-end reads of 50 nt were obtained and mapped on the *M. truncatula* genome version 4.0 (Mt4.0) (Tang et al., 2014). The resulting differential expression profiles were then mined for the closest *M. truncatula* orthologs of a list of known *A. thaliana* ER stress marker genes (Howell, 2013). As such, a set of genes encoding luminal-binding protein 1/2 (BiP1/2; Medtr8g099945), BiP3 (Medtr8g099795), stromal cell derived factor 2 (SDF2; Medtr3g106130), sorbitol dehydrogenase (SDH; Medtr1g025430), calnexin (CNX; Medtr3g098430), UDP-glucose:glycoprotein glucosyltransferase (UGGT; Medtr2g006960), heat-shock protein 70 (HSP70; Medtr3g081170), and protein disulfide isomerase-like 1-1 (PDIL1-1; Medtr3g088220) were found to be significantly upregulated in MKB1^KD^ roots compared to CTR roots, both upon mock and MeJA treatment (**Figure 2A**). Notably, MeJA elicitation itself was also sufficient to trigger an ER stress response in CTR roots, albeit less pronounced (**Figure 2A**), in accordance with the moderately altered ER network structure (**Figure 1**). Together, these data suggest that a transcriptome reminiscent of an ER stress response is not only triggered by loss of MKB1 function but also by JA elicitation.

We further assessed the transcript levels of the putative *M. truncatula* orthologs of the core *A. thaliana* ER stress-related bZIP TFs, *AtbZIP17*, *AtbZIP28* and *AtbZIP60*. Because the bZIP TF gene family is well represented in the *M. truncatula* genome, with at least 81 potential members (Tang et al., 2014), first a phylogenetic analysis for all potential *M. truncatula* bZIP TF genes that were annotated by Wang et al. (2015) was carried out to define the putative *M. truncatula bZIP17*, *bZIP28* and *bZIP60* orthologs. Amino acid sequences of all *M. truncatula* bZIP gene entries, as well as the previously reported *A. thaliana* bZIP TF genes (Dröge-Laser et al., 2018), were retrieved from PLAZA (Van Bel et al., 2018). As previously reported by Dröge-Laser et al. (2018), *AtbZIP17* is part of the group B that also comprises *AtbZIP28* and *AtbZIP49*, whereas *AtbZIP60* is part of the group K as a unique gene. In the *M. truncatula* genome, the bZIP TFs groups B and K are respectively and solely represented by *Medtr7g088890* and *Medtr1g050502* (**Supplementary Figures 3**, **4**). It therefore appears that *M. truncatula* might not have other paralogs for the bZIP TF genes in either group B and K, or, alternatively, they are not annotated yet by the Mt4.0 genome, as for instance is also the case for the *MKB1* gene. However, our results are in accordance with a genome-wide analysis of the bZIP TF gene family previously carried out for six legume genomes (*Glycine max, Phaseolus vulgaris, Cicer arietinum, Cajanus cajan, Lotus japonicas*, and *M. truncatula*), where also only a single ortholog for both group B and K bZIP TFs was encountered in five of the legumes studied (Wang et al., 2015). *G. max* formed a notable exception, with two paralogs in each group, which may be the consequence of a recent whole-genome duplication that *G. max* experienced (Wang et al., 2015).

Subsequent mining of the RNA-Seq data indicated that *bZIP17* gene transcript levels were only significantly upregulated in MKB1^KD^ hairy roots when elicited with MeJA compared to mock treatment of CTR and MBK1^KD^ (~3.2 fold) (**Figure 2B**). However, given that activation and translocation of AtbZIP17 occurs posttranslationally following proteolytic cleavage in the Golgi (Liu et al., 2007a; Zhou et al., 2015; Kim et al., 2018), the lack of transcriptional elicitation of *bZIP17* does not exclude that its activity could be enhanced posttranslationally by loss of MKB1 function or by MeJA elicitation. This possibility was not further investigated, given that this would demand extensive additional experimentation and that the results for *bZIP60* were more indicative of the activation of an ER stress response. Indeed, *bZIP60* transcripts accumulated to significantly higher levels in MKB1^KD^ hairy roots as compared to CTR roots, both in mock (2.6 fold) and MeJA (3.9 fold) conditions (**Figure 2B**). Furthermore, also MeJA treatment could elicit upregulation of *bZIP60* transcript levels particularly in the MKB1^KD^ (2.5 fold) hairy roots (**Figure 2B**). Contrary to bZIP17, ‘activation’ of bZIP60 can be assessed at the transcript level, given that it is regulated by IRE1-mediated splicing (Nagashima et al., 2011). To assess the splicing status of *bZIP60*, qRT-PCR was performed using primers designed to detect the predicted spliced *bZIP60* amplicon. This analysis indicated that the level of spliced *bZIP60* amplicons was increased, both in MKB1^KD^ compared to CTR and following elicitation with MeJA treatment, both in CTR and MKB1^KD^ hairy roots (**Figure 2C**). Together, our transcriptome analysis supports the occurrence of an increased ER stress response, caused by loss of MKB1 function, and to a minor extent also by MeJA elicitation.

### *Medicago truncatula* bZIP17 and bZIP60 can counteract transactivation of TS biosynthesis promoters by TSAR1 and TSAR2

Next, we hypothesized that the two bZIP17 and bZIP60 TFs could negatively regulate TS biosynthesis, hence explaining the TS-specific negative feedback observed in MKB1^KD^ hairy roots (Pollier et al., 2013a). Localization of some form of the bZIPs in the nucleus is expected in this hypothesis. *In silico* analysis of the full-length *M. truncatula* bZIP60 and bZIP17 sequences confirmed the presence of the expected evolutionary splicing (for bZIP60) or protease cleavage (for bZIP17) sites and the respective ER-anchoring transmembrane domains and nuclear localization signals (**Supplementary Figures 5**, **6**), which is in line with the evolutionary conservation of the UPR pathway (Howell, 2021). We designed N-terminal GFP-tagged versions of the full-length and truncated bZIP17 and bZIP60 proteins and expressed those transiently *via* Agroinfiltration of *N. benthamiana* leaves. Though we could not unambiguously determine a single subcellular localization of either the full-length or truncated variants, nuclear localization of both truncated *M. truncatula* bZIP17 and bZIP60 variants was clearly observed (**Supplementary Figure 7**).

**Figure 3:**
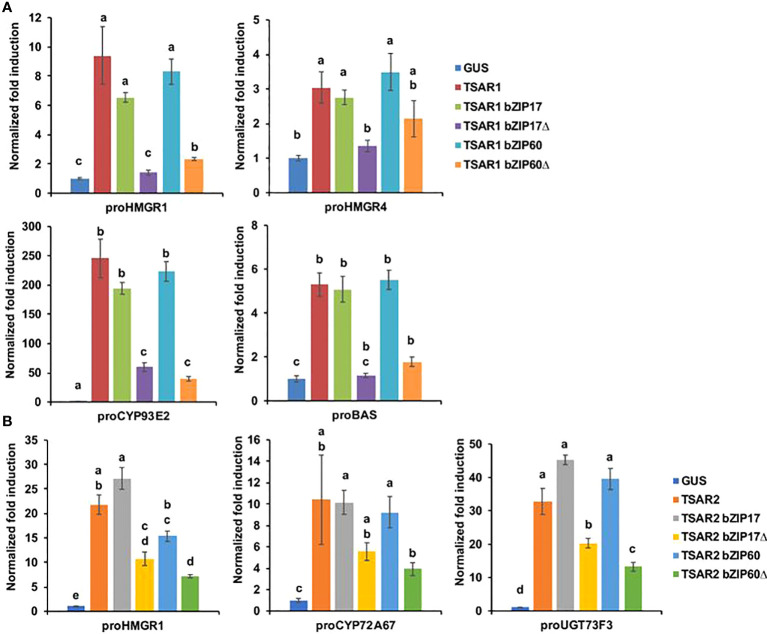
*M. truncatula bZIP17Δ* and *bZIP60Δ* repress transactivation of TS biosynthesis gene promoters by TSAR TFs. **(A, B)** Transient transactivation assays in BY-2 protoplasts using the indicated target promoters fused to the *fLUC* reporter gene and TSAR1 **(A)** or TSAR2 **(B)** as effectors combined with full-length or truncated bZIP17 and bZIP60. The Y-axis shows fold change in normalized fLUC activity relative to the control transfection with *proCaMV35S:GUS.* The error bars designate SE of the mean (n = 8 biological repeats). Different letters indicate statistically significant differences at P < 0.05, as determined by ANOVA, *post hoc* Tukey analysis.

Next, we tested whether bZIP17 and bZIP60 were able to modulate the previously reported transactivation of a set of TS biosynthesis reporter constructs (promoter-fLUC) by TSAR1 or TSAR2 in a transient expression assay in *N. tabacum* Bright Yellow-2 (BY-2) protoplasts (Mertens et al., 2016a). For any of the tested reporter constructs, no effect of both the full-length bZIP17 and bZIP60 or the truncated versions lacking the transmembrane domain, bZIP17Δ and bZIP60Δ, on reporter fLUC activity was observed (exemplified with *proCYP93E2* in **Supplementary Figure 8**). However, the high transactivation of *proHMGR1*, *proHMGR4*, *proβAS*, and *proCYP93E2* by *TSAR1*, as compared to the *GUS* control, was significantly repressed when combined with the truncated *bZIP17Δ* or *bZIP60Δ*, but not with full-length *bZIP17* or *bZIP60* (**Figure 3A**). A similar trend was observed for the transactivation of *proHMGR1*, *proCYP72A67* and *proUGT73F3* by *TSAR2* (**Figure 3B**). Taken together, these data suggest that the truncated *M. truncatula* ER stress regulatory TFs bZIP17Δ and bZIP60Δ can counteract the transactivation of TS biosynthesis genes in *M. truncatula* by TSAR1 and TSAR2 bHLH factors and may therefore be accountable for the TS-specific transcriptional feedback observed in MKB^KD^ hairy roots (Pollier et al., 2013a).

### Functional characterization of *Medicago truncatula* bZIP17 and bZIP60 *in planta*

To further evaluate the *in planta* role of bZIP17/bZIP60 in *M. truncatula*, we generated gain- and loss-of-function hairy root lines for the *bZIP17/bZIP60* genes. Three independently generated root lines expressing the *GUS* gene were used as the control. For *bZIP17*, we generated three independent *M. truncatula* hairy root lines overexpressing *bZIP17Δ* (bZIP17Δ-OE, **Supplementary Figure 9A**). Quantitative reverse transcription-PCR (qRT-PCR) analysis confirmed overexpression of *bZIP17Δ*, though this seemingly did not increase total *bZIP17* transcript levels in an appreciable manner (**Supplementary Figure 9B**). An average fourfold increase of *bZIP60* transcript levels was also observed in those lines. Accordingly, the expression level of the chaperone *BiP1/2* was significantly increased in the bZIP17Δ-OE hairy root lines, in line with previous observations in other species such as *A. thaliana* (Li et al., 2017) and maize (Yang et al., 2013). Only two of the nine tested TS biosynthesis genes, i.e. *CYP716A2* and *UGT73F3*, did show significant differential expression in the three bZIP17Δ-OE lines compared to the CTR (**Supplementary Figure 9C**). Likewise, constitutive ectopic *bZIP17Δ* overexpression did not appear to comprehensively affect MeJA induction of TS biosynthesis genes, because a significant decrease in the MeJA response was only observed for two of the eight tested TS biosynthesis genes, i.e. *BAS* and *CYP716A2* (**Supplementary Figure 10**). Unfortunately, we did not manage to generate lines overexpressing bZIP60Δ, despite several transformation rounds.

**Figure 4:**
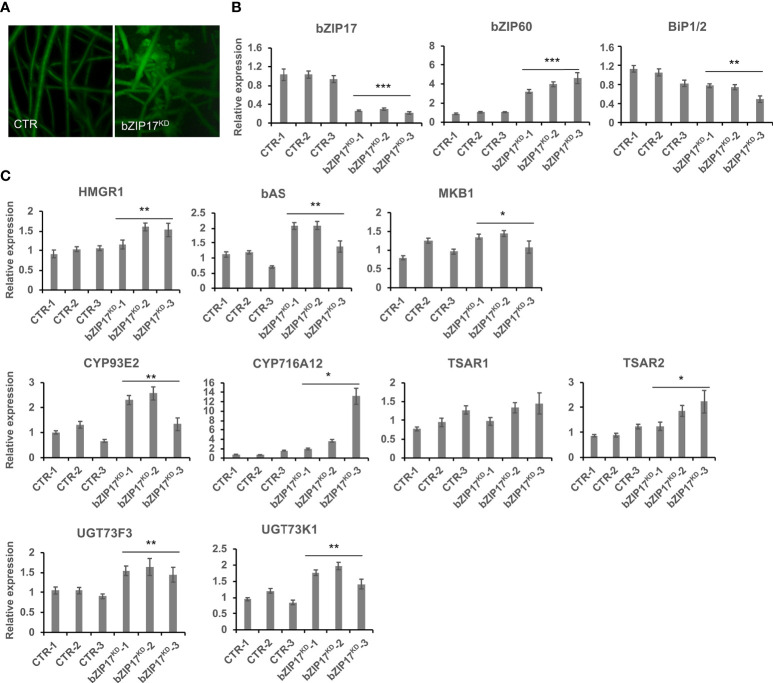
Silencing of *bZIP17* slightly increases TS biosynthesis gene expression in *M. truncatula* hairy roots. **(A)** Morphology of control (CTR) and bZIP17^KD^ hairy roots. **(B, C)** qRT-PCR analysis of *bZIP17*, *bZIP60*, *BiP1/2* genes **(B)** and TS biosynthetic genes **(C)** in three independent CTR and *bZIP17^KD^* hairy root lines. Values in the y-axis represent the expression ratio relative to the normalized transcript levels of CTR lines. The error bars designate SE (n = 3, technical repeats). Statistical significance was determined by a Student’s *t*-test (*P < 0.05, **P < 0.01, ***P < 0.001).

**Figure 5:**
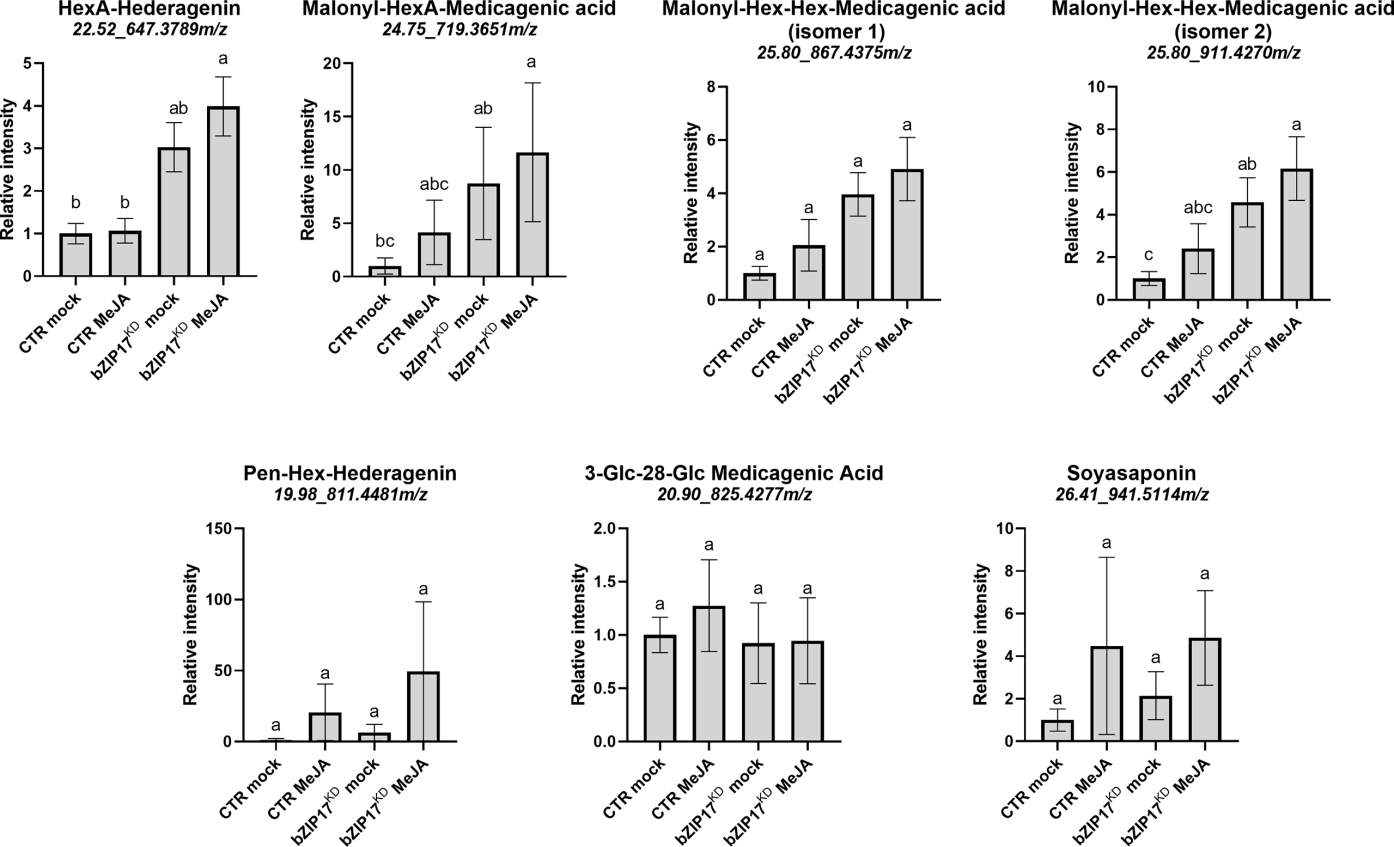
Silencing of *bZIP17* enhances the accumulation of TS in *M. truncatula* hairy roots. Relative accumulation of TS in mock- and MeJA-treated control (CTR) and bZIP17^KD^ hairy roots. Values on the y axis correspond to fold changes relative to the average of the mock-treated control lines. Intensity values were transformed, scaled and analyzed by two-way ANOVA (p ≤ 0.01), categories refer to Tukey’s *post-hoc* test (p < 0.05). For each of the TS, the identity, retention time and m/z value is indicated on top. The small letters represent the categories that refer to Tukey’s post-hoc test (p < 0.05). They indicate significant differences between treatments.

We also managed to generate three independent *bZIP17* and two independent *bZIP60* knock-down lines (bZIP17^KD^ and bZIP60^KD^), all showing approximately a fourfold reduction in *bZIP* expression (**Figure 4B** and **Supplementary Figure 11B**). A notable growth phenotype with a callus-like morphology was observed, especially for bZIP60^KD^ hairy root lines (**Figure 4A** and **Supplementary Figure 11A**), but which is distinct from the caltrop-like MKB1^KD^ phenotype. Furthermore, *bZIP60* transcript levels were significantly increased in the bZIP17^KD^ lines (**Figure 4B**). Conversely, no feedback on *bZIP17* expression was observed in the bZIP60^KD^ hairy root lines (**Supplementary Figure 11B**). Importantly however, the transcript levels of all analyzed TS biosynthesis genes, except those of *TSAR1*, were slightly, but significantly, increased in the bZIP17^KD^ lines (**Figure 4C**). These observations were corroborated with metabolite profiling by liquid chromatography – mass spectrometry (LC-MS) analysis, which demonstrated significantly increased accumulation levels of several of the measured TS in the bZIP17^KD^ lines, both in mock- and MeJA-treated conditions (**Figure 5**). In the bZIP17^KD^ hairy root lines, the effect was less consistent, with only a slight but significant increase in the expression level of *CYP716A12* and *UGT73F3* (**Supplementary Figure 11C**). Unfortunately, we did not manage to generate hairy root lines silencing both *bZIP17* and *bZIP60*, despite several transformation attempts. It is plausible to assume that because of the crucial roles of these bZIP factors for plant physiology, simultaneous loss-of-function of both is not viable. Nonetheless, the data obtained with the *M. truncatula* bZIP17^KD^ lines support the role of at least bZIP17 as a negative attenuator of the TS pathway.

### ER stress inducers repress transcript levels of TS pathway genes

Often, TFs that regulate specialized metabolite biosynthetic pathways are coexpressed with the target genes that encode the enzymes of the pathways, particularly when it concerns JA-modulated pathways (Pauwels et al., 2009; De Geyter et al., 2012). In order to find plant growth or stress conditions in which *M. truncatula bZIP17* and *bZIP60* might show an opposite or correlative expression pattern to those of the TS pathway genes, we mined the *M. truncatula* Gene Expression Atlas (MtGEA; http://bioinfo.noble.org/gene-atlas/) (He et al., 2009). We particularly looked for conditions in which increased *bZIP17* and *bZIP60* expression was observed, in combination with a modulated TS gene expression pattern. Such a situation was encountered for *bZIP17* in roots of *M. truncatula* seedlings grown in the presence of 180 mM NaCl (**Supplementary Figure 12**). This was not unexpected, given that in *A. thaliana*, salt stress was reported to invoke ER stress, for which the action of bZIP17 is needed for the stress coping mechanism (Li et al., 2017). This observation suggested that *in planta* situations, in which altered bZIP60 and bZIP17 activity may modulate TS gene expression, can indeed be encountered.

As far as we could judge, the MtGEA did not seem to contain transcriptome data of other stress or growth conditions with a pronounced effect on *bZIP17/bZIP60* expression or a reported ER stress effect. Because in *A. thaliana*, the reducing agent dithiothreitol (DTT) and the stress hormone salicylic acid (SA) are known to evoke ER stress and to induce the upregulation and activation of *bZIP17* and *bZIP60* (Moreno et al., 2012; Henriquez-Valencia et al., 2015; Li et al., 2017), we decided to analyze *bZIP*, ER stress and TS pathway gene expression in the presence of NaCl, DTT, SA, all in combination or not with MeJA, in CTR *M. truncatula* hairy root lines.

**Figure 6:**
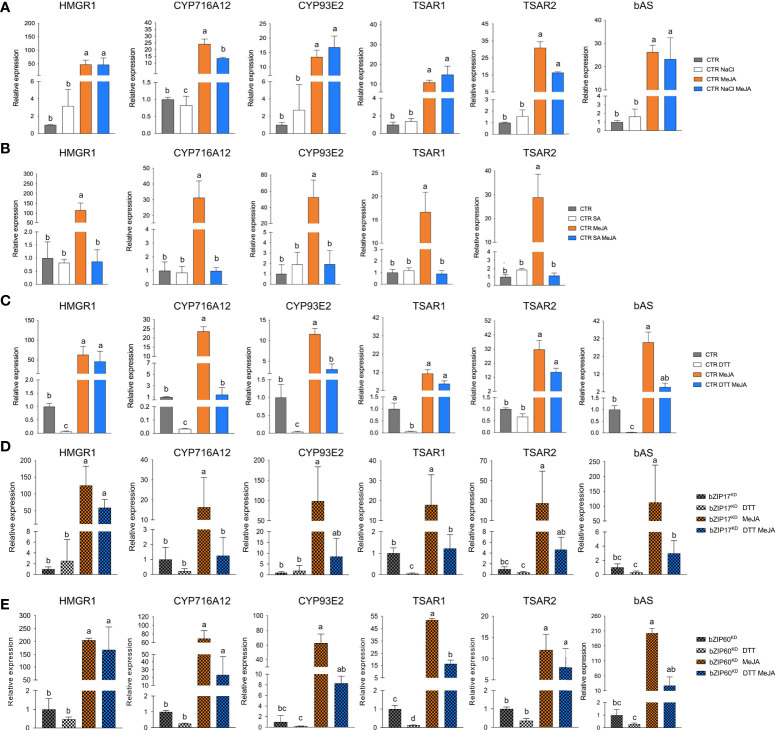
Effects of ER stress on the expression of TS pathway genes in *M. truncatula* hairy roots. **(A-E)** qRT-PCR analysis of TS pathway genes in control (CTR) **(A-C)**, *bZIP17^KD^* **(D)** and *bZIP60^KD^* **(E)** hairy root lines. Hairy roots were treated with 300 mM NaCl **(A)**, 0.5 mM SA **(B)** or 2 mM DTT **(C-E)** for 4 h prior to the 4-h MeJA treatment (5 µM for **(A, C-E)** and 100 µM for **(B)**). Expression ratios were plotted relative to the normalized mock-treated line. The error bars designate SE (n = 3 for **(A-D)** and n = 2 for **(E)**, corresponding to the independent transformed CTR, *bZIP17^KD^* and *bZIP60^KD^* lines generated). Different letters sample indicate statistically significant differences at P < 0.05, as determined by ANOVA, *post hoc* Tukey analysis.

As expected, *bZIP17* and *bZIP60* transcript levels were increased upon NaCl and DTT treatment in the CTR *M. truncatula* hairy roots (**Supplementary Figures 13A, B**). Upon SA treatment, only *bZIP60* transcript levels were significantly increased (**Supplementary Figure 13C**). In all cases, increased *BiP1/2* transcript levels were further indicative of successful ER stress induction by all three stress agents. In most cases, combined application with MeJA aggravated the ER stress as reflected by a further increase in the ER stress gene transcript levels (**Supplementary Figure 13**). Next, we assessed TS pathway gene expression in the CTR line upon different stress treatments. NaCl treatment did not affect the basal expression of TS pathway genes, nor did it interfere with the MeJA elicitation thereof (**Figure 6A**). Different and more interesting trends were observed with the DTT and SA treatments. First, SA had a pronounced inhibitory effect on the MeJA induction of all TS pathway genes tested (**Figure 6B**). Whether this is partly or entirely mediated by increased bZIP activity cannot be judged at this stage however, given that in *A. thaliana* other TFs such as ORA59 or the TF cofactor NONEXPRESSOR OF PATHOGENESIS-RELATED GENES 1 (NPR1) have also been implicated in the SA-mediated suppression of JA signaling (Van Der Does et al., 2013; Nomoto et al., 2021). In this regard, the results obtained with the more specific ER stress agent DTT may be considered more indicative. Indeed, also DTT application significantly suppressed the MeJA elicitation of half of the tested TS pathway genes (**Figure 6C**). To assess whether the DTT-suppressive effect was specific for MeJA elicitation of TS pathway genes, we also assayed expression of known early response JA genes involved in the JA amplification loop (Pauwels et al., 2009). Notably, the transcript levels of *JAZ1*, *LOX*, *MYC2a* and *MYC2b* were significantly increased by DTT treatment (**Supplementary Figure 14**). Moreover, DTT boosted MeJA elicitation of those JA pathway genes. The latter observations can likely be explained by the reported need for a reducing environment for the JA-Ile-induced interaction between COI1 and JAZ1 (Yan et al., 2009). As such, overall, our qRT-PCR analyses indicated that the negative effect of DTT on MeJA elicitation is specific for TS pathway genes.

Finally, we assessed the effect of combined DTT and MeJA application also in the *bZIP17^KD^* and *bZIP60^KD^* hairy root lines. However, no consistent or pronounced differences could be detected with regard to the antagonistic effect that was observed in the CTR line (**Figures 6D, E**). Only MeJA elicitation of *CYP716A12* and *TSAR2* was less pronounced in both knock-down lines, and the suppressive effect of DTT on MeJA elicitation was only attenuated for *CYP716A12*. We assume that either the MeJA effect is capable of overruling the ER stress, or alternatively that the remaining active *bZIP* gene in the knock-down lines can still account for the ER stress effect. Nonetheless, taken together, our data suggest that also *in planta*, ER stress can attenuate the JA response in *M. truncatula* roots, or at least part of it, such as the elicitation of the TS pathway.

### *Catharanthus roseus* bZIP17 and bZIP60 counteract transactivation of monoterpenoid indole alkaloid biosynthesis gene promoters by BIS1

Since the ER stress response is a conserved mechanism in plants and many specialized metabolite biosynthesis pathways are regulated by TFs that bind G-boxes or closely related boxes, we hypothesized that the action of ER stress bZIP factors on the control of specialized metabolite pathways could be conserved across plant species. An obvious model system to explore this is the medicinal plant *C. roseus*, in which different branches of the MIA pathway are controlled by bHLH factors such as the BISs, which are functional orthologs of the TSARs (Van Moerkercke et al., 2015; Van Moerkercke et al., 2016; Mertens et al., 2016b; Schweizer et al., 2018).

To determine the putative orthologs of the *bZIP17* and *b*ZIP*60* genes in *C. roseus*, a BLAST analysis was performed using the AtbZIP17 and AtbZIP60 amino acid sequences as query in the *C. roseus* Functional Genomics Database (croFGD; http://bioinformatics.cau.edu.cn/croFGD/) and Medicinal Plant Genomics Resource (http://medicinalplantgenomics.msu.edu/). The highest significant hits were *CROT021933* and *CROT026761*, respectively, which were confirmed to belong to the bZIP TFs group B and K, respectively, by phylogenetic analysis (**Supplementary Figure 15**). Moreover, *CROT021933* and *CROT026761* shared the same conserved protein sequence motifs with their bZIP TF group members (**Supplementary Figures 15**, **16**).

**Figure 7:**
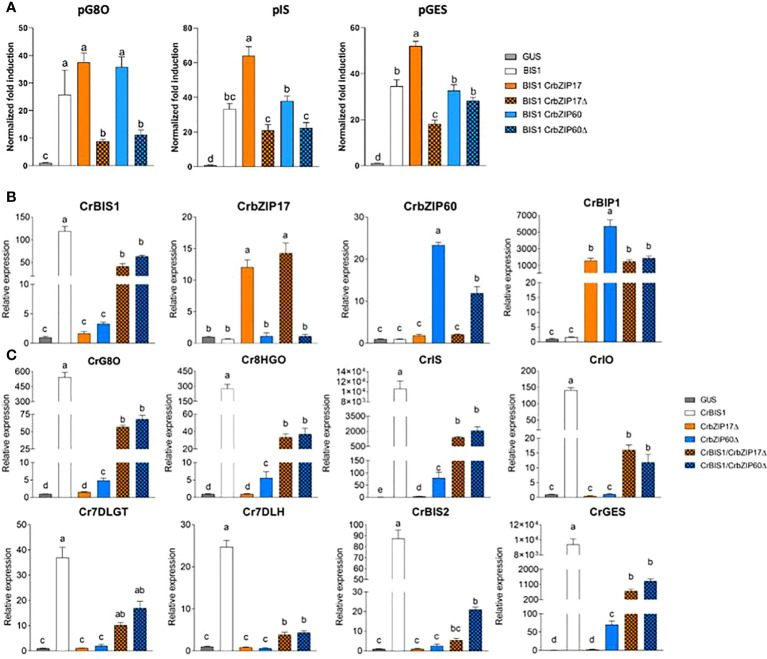
*C. roseus* bZIP17Δ and bZIP60Δ can repress the transactivation of MIA biosynthesis genes by BIS1. **(A)** Transient transactivation assays in BY-2 protoplasts using the indicated target promoters fused to the *fLUC* reporter gene and BIS1 as effector combined with full-length or truncated *C. roseus* bZIP17 and bZIP60. The Y-axis shows fold change in normalized fLUC activity relative to the control transfection with *proCaMV35S:GUS.* The error bars designate SE of the mean (n = 4). Different letters indicate statistically significant differences at P < 0.05 as determined by ANOVA, *post hoc* Tukey analysis. **(B, C)** qRT-PCR analysis of *CrBIS1*, *CrbZIP17*, *CrbZIP60* and *CrBiP1* genes **(B)** and of MIA pathway genes **(C)** in *C. roseus* flower petals transiently overexpressing *CrBIS1*, *CrbZIP17Δ*, *CrbZIP60Δ*, *CrBIS1/CrbZIP17Δ* or *CrBIS1/CrbZIP60Δ* under control of the *CaMV35S* promoter. Control samples were infiltrated with the *pCaMV35S:GUS* construct. The error bars designate SE of the mean (n = 4). Different letters indicate statistically significant differences at P < 0.05, as determined by ANOVA, *post hoc* Tukey analysis.

Next, a transient expression assay was performed in BY2 protoplasts to assess transactivation of a set of promoters from the genes encoding enzymes of the *C. roseus* MIA biosynthesis pathway that are known to be controlled by the BIS TFs (Van Moerkercke et al., 2015; Mertens et al., 2016b; Van Moerkercke et al., 2016; Schweizer et al., 2018), including *geraniol 8-oxidase* (*G8O*), *geraniol synthase* (*GES*) and *iridoid synthase* (*IS*), by the *C. roseus* BIS1 TF in combination with *C. roseus* CrbZIP17, CrbZIP60 or the truncated versions thereof (CrbZIP17Δ and CrbZIP60Δ). And indeed, the transactivation mediated by BIS1 of *proG8O* and *proIS* was compromised in the presence of the truncated *CrbZIP17Δ* and *CrbZIP60Δ*, but not of the intact *CrbZIP17* and *CrbZIP60* (**Figure 7A**). In the case of *proGES*, counteraction of BIS1-mediated transactivation was only observed with *CrbZIP17Δ* (**Figure 7A**). Together, these data suggest that translocated ER stress response bZIP TFs can counteract the BIS1-mediated transcriptional activation of MIA biosynthesis genes in *C. roseus*.

To further corroborate this, we also assessed the effect of the *C. roseus* bZIPs in *C. roseus in planta*, in particular through an *A. tumefaciens*-assisted *C. roseus* flower infiltration platform that was previously successfully used as an expression system to (co-)express TF(s) and thereby screen for novel MIA biosynthesis regulators (Schweizer et al., 2018). Here, we transiently transformed *C. roseus* flowers with overexpression cassettes for *BIS1*, *CrbZIP17Δ*, and *CrbZIP60Δ*, as well as the double combinations *BIS1/CrbZIP17Δ and BIS1/CrbZIP60Δ.* qRT-PCR analysis confirmed overexpression of all TF genes (**Figure 7B**). In support of the functionality of the bZIP TFs, the expression level of the chaperone *BiP1* was significantly increased in all the bZIP17Δ-OE and bZIP60Δ-OE samples (**Figure 7B**). Likewise, in line with previous observations (Van Moerkercke et al., 2015; Van Moerkercke et al., 2016; Schweizer et al., 2018), overexpression of *BIS1* strongly upregulated all known BIS targets of the MIA pathway genes tested (**Figure 7C**). Finally, as anticipated, the combinatorial overexpression of *CrbZIP17Δ* or *CrbZIP60Δ* could significantly counteract the BIS1-mediated transcriptional activation of all MIA pathway genes tested (**Figure 7C**). Taken together, our findings suggest that interference by ER stress bZIP TFs in the attenuation of JA-dependent terpene biosynthetic pathways might be widespread in the plant kingdom.

## Discussion

The ERAD-type E3 ubiquitin ligase MKB1 has previously been reported to manage TS biosynthesis in *M. truncatula* by controlling HMGR stability (Pollier et al., 2013a). Silencing of *MKB1* in *M. truncatula* MKB1^KD^ hairy roots results in an aberrant caltrop-like morphology, increased accumulation of monoglycosylated TS, decreased accumulation of higher glycosylated TS, and a specific downregulation of TS biosynthesis gene expression. Pollier et al. (2013a) speculated that this TS-specific transcriptional response might constitute a negative feedback loop to cope with the ectopic accumulation of bioactive monoglycosylated saponins.

Intrigued by this anomaly, we explored the MKB1^KD^ hairy root phenotype by additional microscopic and transcriptomic analyses. These combined analyses pointed to an ER stress response, reflected by an altered ER network structure and increased transcript levels of *M. truncatula* orthologs of known *A. thaliana* ER stress marker genes. Because in *A. thaliana*, and conceivably plants in general, the ER stress response can be mediated by two signaling branches, both depending on bZIP TFs, AtbZIP17/AtbZIP28 and AtbZIP60, respectively, that translocate from the ER to the nucleus in ER stress conditions, we speculated that the TS-specific feedback in MKB1^KD^ hairy roots may be mediated by the action of the *M. truncatula* orthologs of these bZIP TFs. Indeed, subsequent functional analysis confirmed that the truncated versions of *M. truncatula* bZIP60 and bZIP17 can localize to the nucleus and interfere with TSAR1/TSAR2-mediated transactivation of TS gene promoters. We therefore speculate that this mechanism could be imposed by plants to attenuate or fine-tune the biosynthesis of TS under particular stress conditions.

### What is the molecular mode of action by which the bZIP TFs repress TSAR-induced TS gene expression?

It remains to be determined how the bZIP and TSAR TFs interact to modulate TS biosynthesis. It has been demonstrated in *A. thaliana* that under non-stressed conditions, the activity of bZIP28 is inhibited by elongated hypocotyl 5 (HY5), another bZIP TF (Nawkar et al., 2017), by competition for binding to the G-box element (CACGTG) displayed within the ER stress response element (ERSE) motifs in the promoters of the UPR genes. Under ER stress conditions, HY5 undergoes proteasomal degradation, releasing the competition and enabling bZIP28 to bind to the ERSE motifs and activate the UPR (Nawkar et al., 2017). A similar scenario has been reported for JA-inducible genes by Van Der Does et al. (2013) in *A. thaliana*, where SA can suppress JA signaling downstream of the JA receptor by targeting GCC promoter motifs *via* the TF octadecanoid-responsive Arabidopsis AP2/ERF domain protein 59 (ORA 59). In *C. roseus*, the production of MIAs is regulated by the bHLH TF CrMYC2 (Zhang et al., 2011; Paul et al., 2017; Schweizer et al., 2018). Overexpression of *CrMYC2* induces expression of the genes encoding bZIP G-box binding factors (GBFs), resulting in a reduced alkaloid accumulation in *C. roseus* hairy roots (Sui et al., 2018). Given that CrGBF1 can bind the same *cis*-element (T/G-box) as CrMYC2 in MIA biosynthesis gene promoters and that CrGBFs can dimerize with CrMYC2, it has been suggested that CrGBF TFs can antagonize CrMYC2 by competitive binding to the T/G-box, and/or by forming a heterodimeric complex, preventing CrMYC2 from binding its target promoters (Sui et al., 2018). Accordingly, we hypothesize a mechanism in *M. truncatula*, in which induction of TS biosynthesis genes by the bHLH TFs TSAR1 and TSAR2 could be antagonized by bZIP17 and bZIP60, plausibly either by competitive binding to the promoters or by the formation of a protein complex that would impede TSAR TF transcriptional activity.

**Figure 8:**
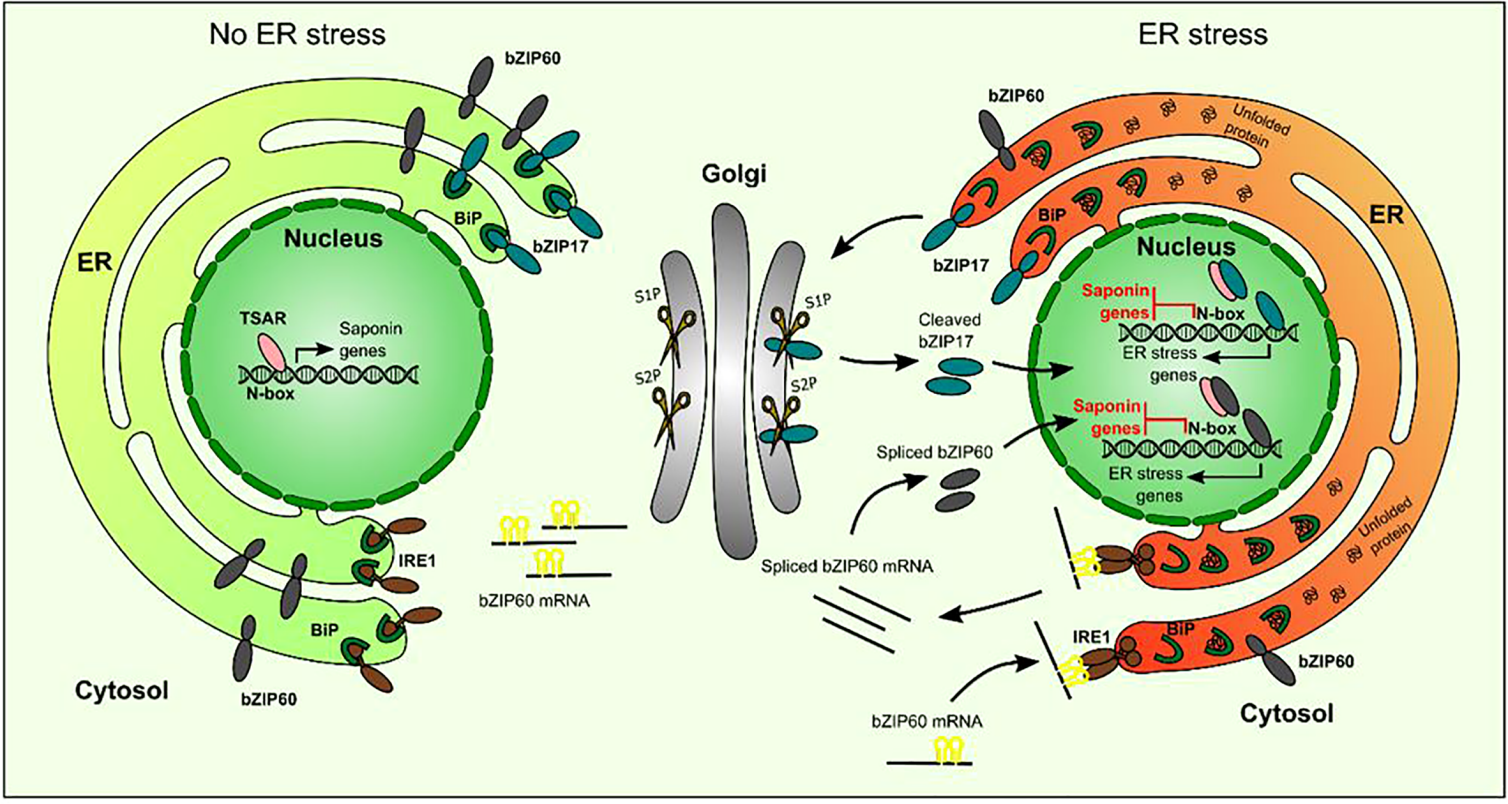
Hypothetical model of action of bZIP17 and bZIP60 and TSAR under normal and ER stress conditions. Under non-ER stress conditions, TSAR can bind to the N-box present in the promoters of TS biosynthesis genes and activate their expression. This can occur both in the absence and presence of JAs. Under ER stress conditions, unfolded proteins accumulate and bind to BiP in the ER. This triggers IRE1 to perform unconventional splicing of *bZIP60* mRNA as well as the release of bZIP17 from the ER and its cleavage by proteases in the Golgi apparatus. The resulting active forms of bZIP17 and bZIP60 translocate to the nucleus, where they can transactivate the expression of ER stress-responsive genes but also hinder the TSARs and thereby repress the transactivation of TS biosynthesis gene expression. TSAR, triterpene saponin biosynthesis activating regulator; bZIP, basic leucine zipper TF; BiP, binding protein chaperone; IRE1, inositol-requiring enzyme 1; S1P, site-1 protease; S2P, site-2 protease.

ERSE-like *cis-*elements, the targets of the ER stress response bZIPs, are present in several of the TS gene promoters. Notably, in the minimal *CYP93E2* promoter region that contains N-box motifs (5´-CACGAG-3´) that are necessary and sufficient for TSAR-mediated transactivation (Mertens et al., 2016a), this ERSE-like box (5´-ATTCGACCACG-3´) overlaps with one of the N-boxes, making this promoter a plausible target for inhibitory crosstalk. However, given that partial substitution of the ERSE-like motif would also affect the N-box, and that the latter reduces the capacity of TSAR1 to transactivate this promoter fragment (Mertens et al., 2016a), we could not unambiguously assess this hypothesis. Likewise, we pursued several protein–DNA and protein–protein interaction methods, including yeast one-hybrid to investigate interactions of bZIPs with TS gene promoters, and yeast two-hybrid and bimolecular fluorescence complementation in agro-infiltrated *N. benthamiana* leaves, to assess possible TSAR–bZIP protein interactions, but none of these assays yielded conclusive results. Hence, our model on how ER stress-related bZIPs hinder TSAR1 transcriptional activity, and, by consequence, transactivation of TS biosynthesis genes (**Figure 8**), remains speculative with regard to the exact *modus operandi*.

### Physiological relevance and evolutionary conservation of the suppressive effect of ER stress-induced bZIP factors on JA-inducible and bHLH TF-mediated elicitation of terpene biosynthesis in plants

The molecular mechanisms of ER stress signaling have been well studied over the last years, at least in *A. thaliana*. Many studies made use of compounds such as tunicamycin, which blocks N-linked glycosylation, or the reducing agent DTT, as artificial ER stress inducers. Exploiting such ER stress agents, in particular DTT, we could further support the inverse correlation between TS expression and levels of ER stress, as DTT treatment repressed the expression, particularly in mock conditions and to a lesser extent in the presence of MeJA, of several TS genes encoding both pathway enzymes and the regulatory TSARs themselves in *M. truncatula* hairy root lines.

There is also increasing knowledge of the physiological conditions upon which ER stress is induced ‘naturally’ in plants. For instance, salt and heat stress have been shown to activate an overall ER stress response in *A. thaliana* (Gao et al., 2008; Deng et al., 2011; Tang et al., 2012; Li et al., 2017). Furthermore, also SA was reported to induce both IRE1/bZIP60 and bZIP17/bZIP28 branches of ER stress signaling in *A. thaliana* (Nagashima et al., 2014), although how is still under debate. It has been reported that SA treatment activates phosphatidylinositol 4-kinase (PI4K), thereby increasing PI phosphates (PIPs) in the Golgi membrane (Krinke et al., 2007). Contrarily, inhibition of PI4K activity leads to a decrease in PIPs and inhibition of *BIP3* induction upon SA treatment (Krinke et al., 2007). Krinke et al. (2007) speculated that upon SA treatment, the phospholipid content in the ER membrane system and traffic within is changed, thereby activating ER-localized ER stress sensors in *A. thaliana*. During SA defense, the transcriptional cofactor NPR1 is converted from an oligomer into a monomer, which leads to the expression of *pathogenesis-related protein 1* (*PR1*) gene family members (Wang et al., 2005). Recently, NPR1 has also been shown to be able to interact with bZIP28 and bZIP60 and to suppress the UPR, independently from SA (Lai et al., 2018). Furthermore, Meng et al. (2017) showed that the constitutive expresser of pathogenesis‐related genes 5 (CPR5), a negative modulator of SA, inhibits both the SA-dependent IRE1/bZIP60 and the ER stress‐induced bZIP28/IRE1–bZIP60 branches, favoring the growth of plants. Many studies reported both on antagonistic (Rojo et al., 2003; Bostock, 2005; Beckers and Spoel, 2006) and synergistic interactions between SA and JA in plants (Schenk et al., 2000; Van Wees et al., 2000; Mur et al., 2006). SA appears to confer resistance to biotrophic pathogens, whereas JA to insect herbivory and necrotrophic pathogens (Pieterse et al., 2001; Bostock, 2005; Stout et al., 2006). This regulatory mechanism could offer the plant a way to activate specific stress response pathways, while repressing others, and, thus contribute to the homeostasis of the cell and/or the appropriate defense response. As indicated above, one mechanism by which SA could antagonize JA action is through the targeting of GCC promoter motifs *via* the TF ORA59 in *A. thaliana* (Van Der Does et al., 2013). More recently, the immune cofactor NPR1 was found to be recruited to JA-responsive promoter regions that are co-occupied by a transcription complex consisting of MYC2 and the MED25 Mediator subunit. In the presence of SA, NPR1 physically associates with MYC2 and inhibits transcriptional activation by disrupting MYC2’s interaction with MED25 (Nomoto et al., 2021). Our study suggests that another mechanism of SA–JA antagonism could involve ER stress and the bZIP factors involved therein, but this hypothesis still needs further support with additional experimentation. Nonetheless, such an additional mechanism involving the ER stress machinery could offer plants a means to activate stress response or defense pathways in a stress-specific manner, allowing plants to distinguish between biotic and abiotic stress, or between different pathogens and other attackers. An SA-induced ER stress response could be a mechanism to restrain JA signaling, or at least one of its outputs, namely the elicitation of terpene and/or other specialized metabolite pathways. Given the fact that we observed an antagonism between ER stress-inducible bZIP TFs and JA-inducible bHLH factors in two distinct species, *M. truncatula* and *C. roseus*, which each produce a species-specific compendium of JA-inducible terpene metabolites, this mechanism may be widespread in the plant kingdom. Notably, activation of ER stress and the UPR by JA treatment was recently demonstrated in tomato (Czékus et al., 2020), indicating that (complex) interplay between ER stress and JA signaling may be common in the plant kingdom. As such, the latter as well as our study may open an avenue for new research on how plants fine-tune their interaction with an ever-changing and often hostile environment.

## Data availability statement

The data presented in the study are deposited in the ArrayExpress database, accession number E-MTAB-11668.

## Author contributions

AG conceived the project, original screening and research plans. AG supervised the experiments. BR, M-LE, MC, EL, EC, CW, EVH, RC and MP performed the experiments. BR, M-LE, MC, EL, EC, CW, EVH and AG designed the experiments and analyzed the data. BR, M-LE and AG wrote the article with contributions of all the authors. AG agrees to serve as the author responsible for contact and ensures communication. All scientists who have contributed substantially to the conception, design or execution of the work described in the manuscript are included as authors, in accordance with the guidelines from the Committee on Publication Ethics (COPE) (http://publicationethics.org/resources/guidelines). All authors agree to the list of authors and the identified contributions. All authors contributed to the article and approved the submitted version.
