# Corrigendum: Interference between ER stress-related bZIP-type and jasmonate-inducible bHLH-type transcription factors in the regulation of triterpene saponin biosynthesis in *Medicago truncatula*

**DOI:** 10.3389/fpls.2022.1103943

**Published:** 2022-12-06

**Authors:** Bianca Ribeiro, Marie-Laure Erffelinck, Elia Lacchini, Evi Ceulemans, Maite Colinas, Clara Williams, Evelien Van Hamme, Rebecca De Clercq, Maria Perassolo, Alain Goossens

**Affiliations:** ^1^ Department of Plant Biotechnology and Bioinformatics, Ghent University, Ghent, Belgium; ^2^ VIB Center for Plant Systems Biology, Ghent, Belgium; ^3^ VIB Bio Imaging Core, Ghent, Belgium; ^4^ Cátedra de Biotecnología, Departamento de Microbiología, Inmunología y Biotecnología, Facultad de Farmacia y Bioquímica, Universidad de Buenos Aires, Buenos Aires, Argentina; ^5^ Instituto de Nanobiotecnolog´ıa (NANOBIOTEC), Consejo Nacional de Investigaciones Científicas y TécnicasUniversidad de Buenos Aires, Buenos Aires, Argentina

**Keywords:** triterpene, saponin, *Medicago catharanthus*, *catharanthus*, jasmonate, endoplasmic reticulum, bZIP, basic helix-loop-helix

In the published article, there was an error in the present address of Maite Colinas. Instead of “Institute of Molecular Plant Biology, ETH Zurich, Zurich, Switzerland”, it should be “Department of Natural Product Biosynthesis, Max Planck Institute for Chemical Ecology, Jena, Germany”.

The authors apologize for this error and state that this does not change the scientific conclusions of the article in any way. The original article has been updated.

