# Supplementary material for: Interference between ER stress-related bZIP-type and jasmonate-inducible bHLH-type transcription factors in the regulation of triterpene saponin biosynthesis in Medicago truncatula
Source: Front Plant Sci. 2022 Sep 30;13:903793. doi: 10.3389/fpls.2022.903793 (PMC9562455; doi:10.3389/fpls.2022.903793)
Supplement: Supplementary file 1 [file DataSheet_1.docx]

**Supplementary Material**

**Supplementary Table 1.** Primers used in this study.

| **Primer name** | **Primer sequence (5'-3')** |
| --- | --- |
| **For cloning** |  |
| Mtbzip17Δ attb1 Fw | GGGGACAAGTTTGTACAAAAAAGCAGGCTCCATGGCTGATTCAATTCTCACCTTTC |
| Mtbzip17Δ attb2 Rv | GGGGACCACTTTGTACAAGAAAGCTGGGTCTCAAACTTTCTTAGTTTTCACCTCACTCTTC |
| Mtbzip60Δ attb1 Fw | GGGGACAAGTTTGTACAAAAAAGCAGGCTCCATGGACACTTTCGAACCCC |
| Mtbzip60Δ attb2 Rv | GGGGACCACTTTGTACAAGAAAGCTGGGTCTCAAGACTCCTGCATGGTCATGG |
| Mtbzip60 full-length attb1 Fw | GGGGACAAGTTTGTACAAAAAAGCAGGCTCCATGGACACTTTCGAACCCCAAATCG |
| Mtbzip60 full-length attb2 Rv | GGGGACCACTTTGTACAAGAAAGCTGGGTCTCATAACATGATAAAATCAAATTTC |
| Mtbzip17 full-length attb1 Fw | GGGGACAAGTTTGTACAAAAAAGCAGGCTCCATGGCTGATTCAATTCTCAC |
| Mtbzip17 full-length attb2 Rv | GGGGACCACTTTGTACAAGAAAGCTGGGTCTCAAACATAAGCTGTTACC |
| CrbZIP60 full-length attb1 Fw | GGGGACAAGTTTGTACAAAAAAGCAGGCTTAATGACCGATTTTACGAATCTCGG |
| CrbZIP60 full-length attb2 Rv | GGGGACCACTTTGTACAAGAAAGCTGGGTACTATAAGAGACAGATGAGCATTAAGCA |
| CrbZIP60Δ attb2 Rv | GGGGACCACTTTGTACAAGAAAGCTGGGTATCAAGACTCCTGCTTGGTCTTGGAAGC |
| CrbZIP17 full-length attb1 Fw | GGGGACAAGTTTGTACAAAAAAGCAGGCTTAATGGCTAACACAGCGGTGG |
| CrbZIP17 full-length attb2 Rv | GGGGACCACTTTGTACAAGAAAGCTGGGTATCAGGTAGTAACTAAATGAGGACCA |
| CrbZIP17Δ attb2 Rv | GGGGACCACTTTGTACAAGAAAGCTGGGTATCACACCTTCTTAGTTTTTCCCTCACT |
| **For RT-qPCR** |  |
| MtHMGR1 Fw | CAGGATTCACAGTCACAACAAC |
| MtHMGR1 Rv | GTAGACGAAGGAAGCGATGAG |
| MtCYP93E2 Fw | ATTGGTGAACTTCTTGGTG |
| MtCYP93E2 Rv | TCCTTCTTCCTATCACTACC |
| MtCYP716A12 Fw | AAGGGACAGCATCACCAACAC |
| MtCYP716A12 Rv | CGCCGAGATATTTGACAAGGAAAG |
| MtBAS1 Fw | AATTTCATCTCCGAGGCAGTT |
| MtBAS1 Rv | CGTGGAAGAACACATCCATCT |
| MtMKB1 Fw | CTGTGGTCACCTGTATTG |
| MtMKB1Rv | CCTGTAGTTTATTGGATTCG |
| MtELFa Fw | ACTGTGCAGTAGTACTTGGTG |
| MtELFa Rv | AAGCTAGGAGGTATTGACAAG |
| Mt40S Fw | GCCATTGTCGAATTTGATGCTG |
| Mt40S Rv | TTTTCCTACCAACTTCAAAACACCG |
| MtTSAR1 Fw | TGTGGTGATTATGAGAATGTTGA |
| MtTSAR1 Rv | AAGGTATGTGGCTGGAGAA |
| MtTSAR2 Fw | TCAGTTTCAAGTTCCATCTT |
| MtTSAR2 Rv | AATCGGTTGGAGACAATG |
| MtJAZ1 Fw | AGAGCCAGCCTTCATTTCCT |
| MtJAZ1 Rv | CTCCAGAAACCGATGAAGCG |
| MtLOX Fw | TGGAACTTTGAAGCCGTTGG |
| MtLOX Rv | GTTGGTGATGGCAAGAGTCG |
| MtMYC2a Fw | ACTTCGAGCGGTTGTTCCTA |
| MtMYC2a Rv | TTGTGGTGGCTGATGAGACT |
| MtMYC2b Fw | CTACACCTCTACTCGGCTGG |
| MtMYC2b Rv | GGTGCCGTTGACAAAGGATT |
| Mtbzip17 Fw | AGGCTGTGATCTGAAGAGGAAG |
| Mtbzip17 Rv | TTGTTGAAATTGCTGTTGTTCC |
| Mtbzip60 Fw | CCCTATCCCTCTATCGGATGAT |
| Mtbzip60 Rv | GTTCCTCATCTGCCTAATTTGC |
| MtBiP1/2 Fw | GTGTCATTGCTGGTTTGAATGT |
| MtBiP1/2 Rv | ACCTCCAAGATCAAAGACGAGA |
| MtIRE1 Fw | GTTGCTGTCAAACGTCTTGTTC |
| MtIRE1 Rv | CATATTCCACACCATACCATCG |
| CrbZIP17 Fw | TTCGTTTTGCCTGGATCCGA |
| CrbZIP17 Rv | CAGACACATGGGGGAACTCC |
| CrbZIP60 Fw | AAGGGCGGGAATCAGCTTC |
| CrbZIP60 Rv | TCCTCATCTGCCTTTTGCGT |
| CrGES Fw | GGGCAAGGTGTCACTGAAGA |
| CrGES Rv | CCCAAATCATCCCAAAGGCG |
| CrG10H Fw | TGCTTTGGCATTCAAACCCG |
| CrG10H Rv | GCAAATTCTTCGGCCAGCAC |
| Cr8HGO Rv | GCATTCCTGGACACGAAGGA |
| Cr8HGO Fw | GCATTCCCCACATTCTCCCA |
| CrIS Fw | AGTAGTAGGAGTCACCGGCA |
| CrIS Rv | CTTGCCACGCCGTATACCTT |
| CrIO Fw | TCAAGTCCAAATACGGGCCG |
| CrIO Rv | CGGCGGAAACTGCATTTTGA |
| Cr7DLGT Fw | GCAGAGGGAGTTCTCAAGGC |
| Cr7DLGT Rv | TGGCCAATGCACATTCTTGG |
| Cr7DLH Fw | GGTCTTGAATCACCCTGCCA |
| Cr7DLH Rv | TGGGACACCAGCCACAATAC |
| CrBiP1 Fw | GTGGAATCCTGAGTGGCGAA |
| CrBiP1 Rv | TGACCCCTCCTACCGTTTCA |
| CrBIS1 Fw | ATGGAATCAGTGGTGCTAGTGA |
| CrBIS1 Rv | TTCAATTTCAGGGAGCTGTGAC |
| CrBIS2 Fw | CCATTATTGCTGAGATGGAGAA |
| CrBIS2 Rv | CTTCATTCAGTCTGCCATTGGT |


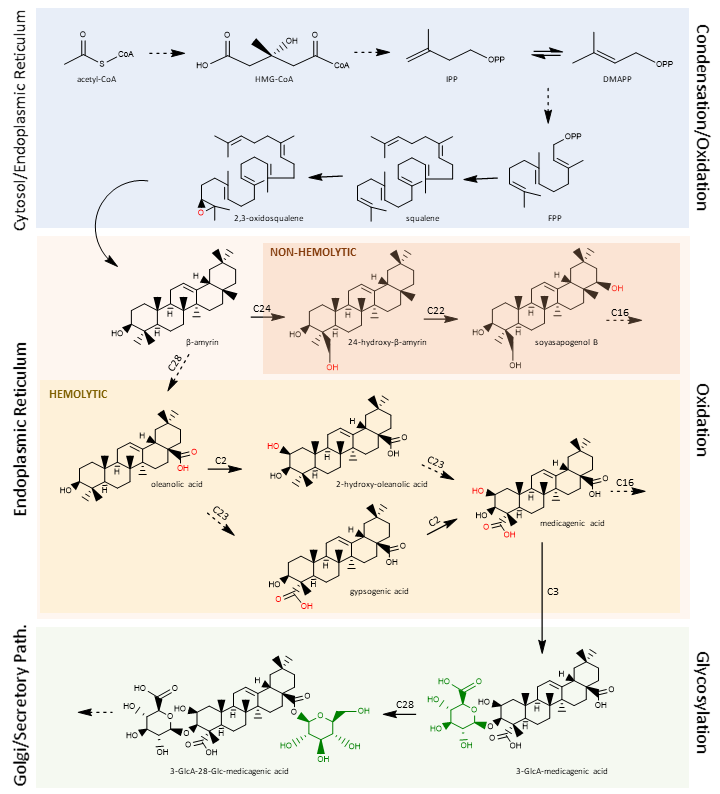


**Supplementary Figure 1.** Schematization of the TS biosynthetic pathway in *M. truncatula.* Starting from the cytosol, acetyl-CoA is used to produce the basic building blocks of all terpenes, namely isopentenyl pyrophosphate (IPP) and its isomer dimethylallyl pyrophosphate (DMAPP). Through several intermediates, these compounds will be ultimately condensed to synthesize squalene, the linear C30 that will be oxidized to its epoxy-form 2,3-oxidosqualene, the last common precursor with the ubiquitous phytosterols. For TS biosynthesis, 2,3-oxidosqualene is cyclized into β-amyrin, the backbone (aglycone) of most TS in *M. truncatula*. Oxidation of β-amyrin at specific positions of the carbon skeleton catalyzed by ER-bound P450 enzymes will confer haemolytic (C-28 oxidation) or non-haemolytic (C-24 oxidation) properties to the TS. Along the haemolytic branch, C-28 oxidation will be followed by either C-2 or C-23 oxidation, leading to the production of medicagenic acid. Later, medicagenic acid will be glycosylated by UDP-glycosyltransferase enzymes along the secretory pathway, conferring amphipathic features to the otherwise largely apolar backbone and determining its biological properties. Dashed and solid arrows indicate multiple- or single-step reactions, respectively. Red and green moieties highlight newly added groups during oxidation and glycosylation reactions, respectively.

**Supplementary Figure 2.** Determination of the minimal MeJA concentration with which TS pathway gene expression is still induced. qPCR analysis of *HMGR1, βAS, MKB1, CYP93E2* and *CYP716A12* genes in control *M. truncatula* hairy root lines. Hairy roots were treated with different concentrations of MeJA for 4 h. The error bars designate SE (n = 3).


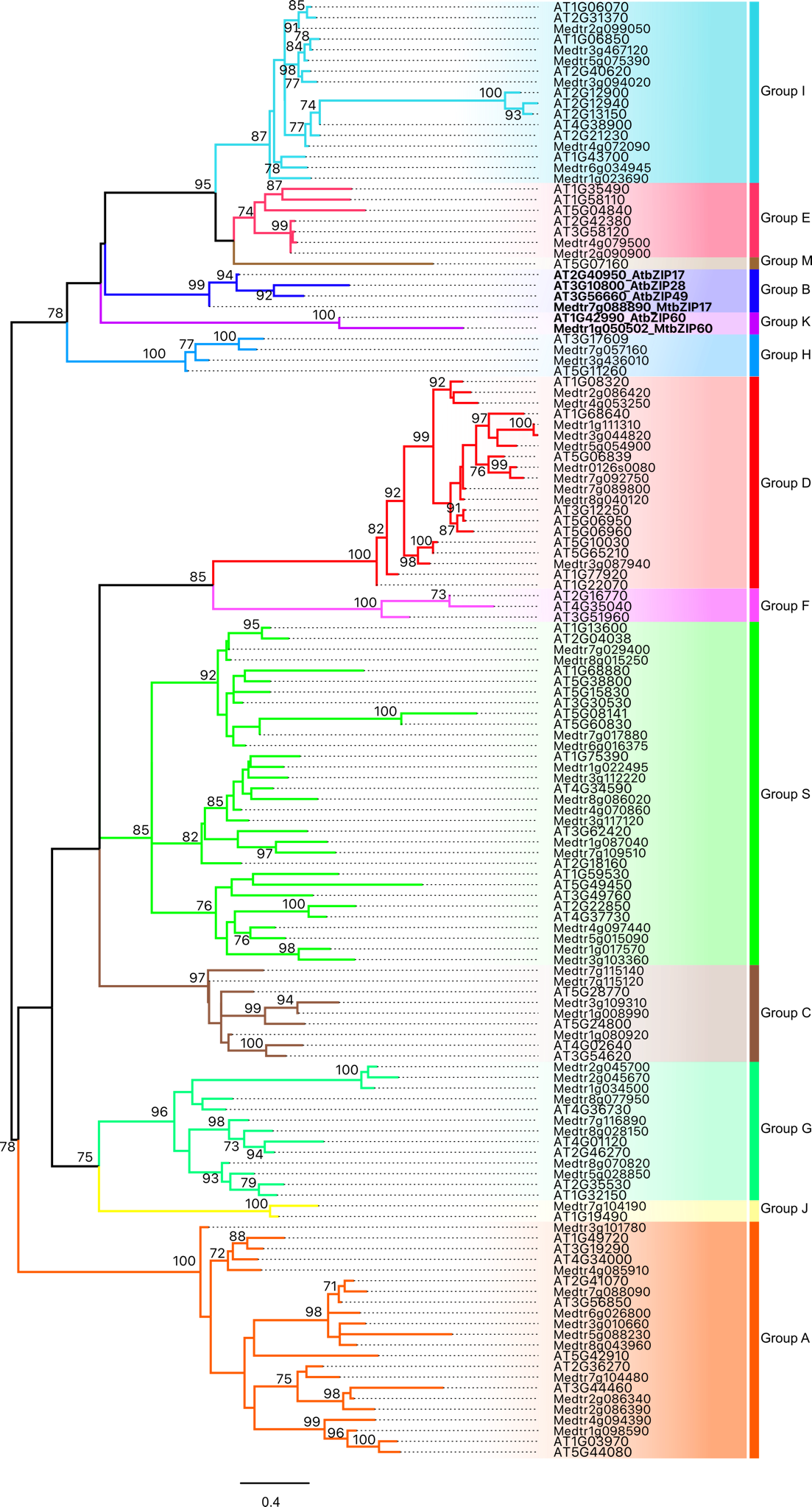

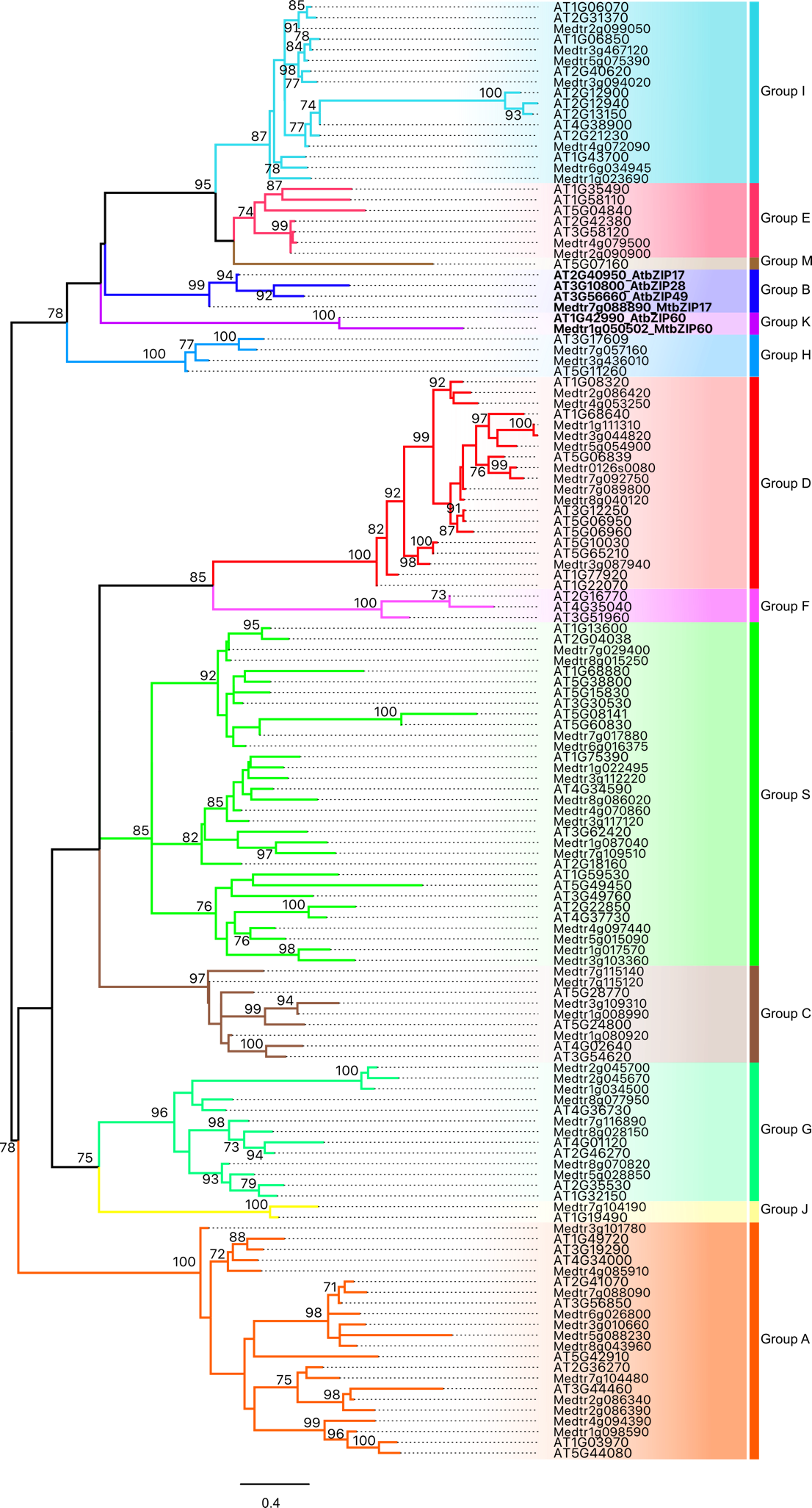


**Supplementary Figure 3.** Phylogenetic analysis of *A. thaliana* and *M. truncatula* bZIP transcription factors. Maximum likelihood phylogenetic tree was generated using the best-fit model LG+R4 with 1000 bootstrap replicates using IQTREE. Bootstrap values greater than 70 are given at the nodes. The scale bar indicates number of changes. The grouping of bZIPs was based on Dröge-Laser et al. (2018). The alignment used to build the phylogenetic tree can be found in Supplementary Figure 4.

**
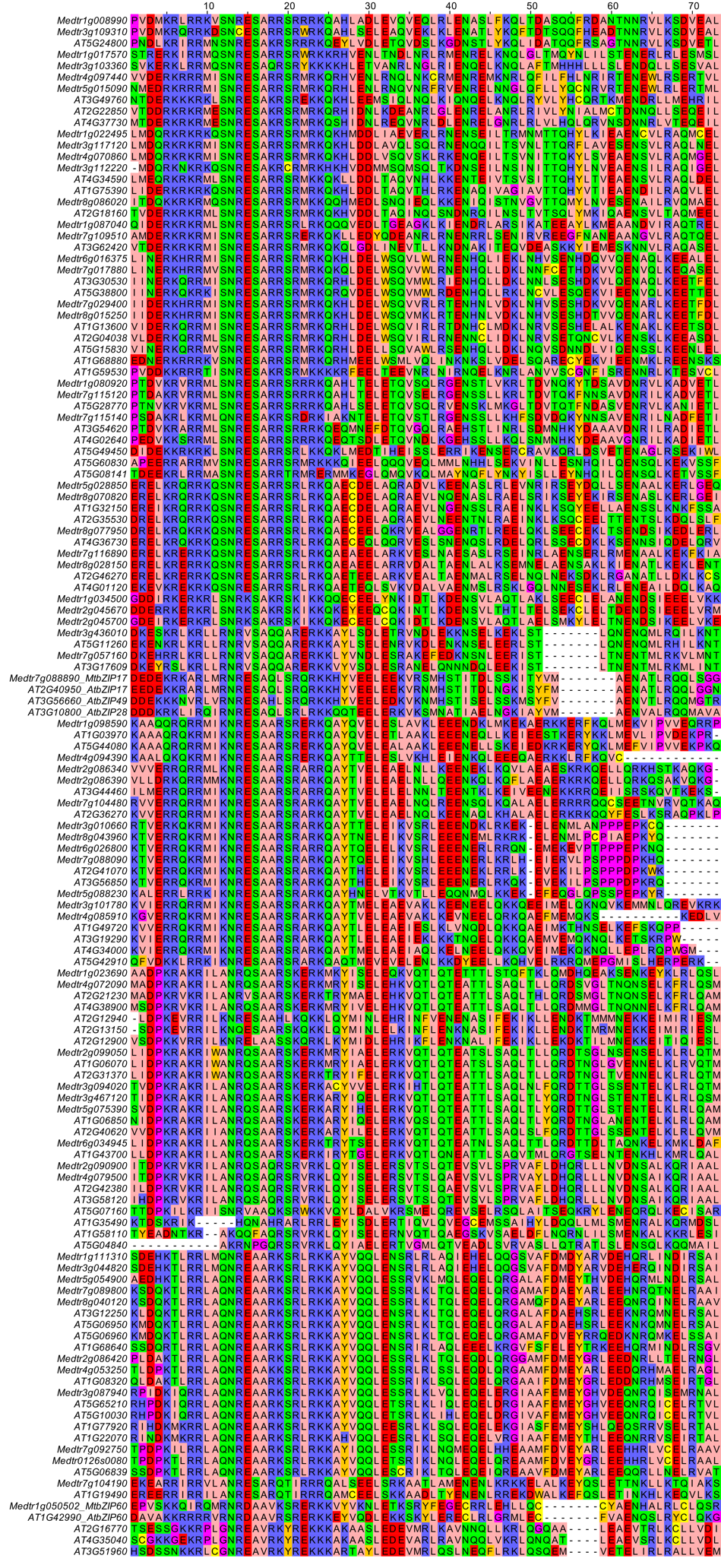
**

**Supplementary Figure 4.** Sequence alignment used for the phylogenetic analysis of *A. thaliana* and *M. truncatula* bZIP transcription factors. Alignment was carried with the bZIP domain only.

**
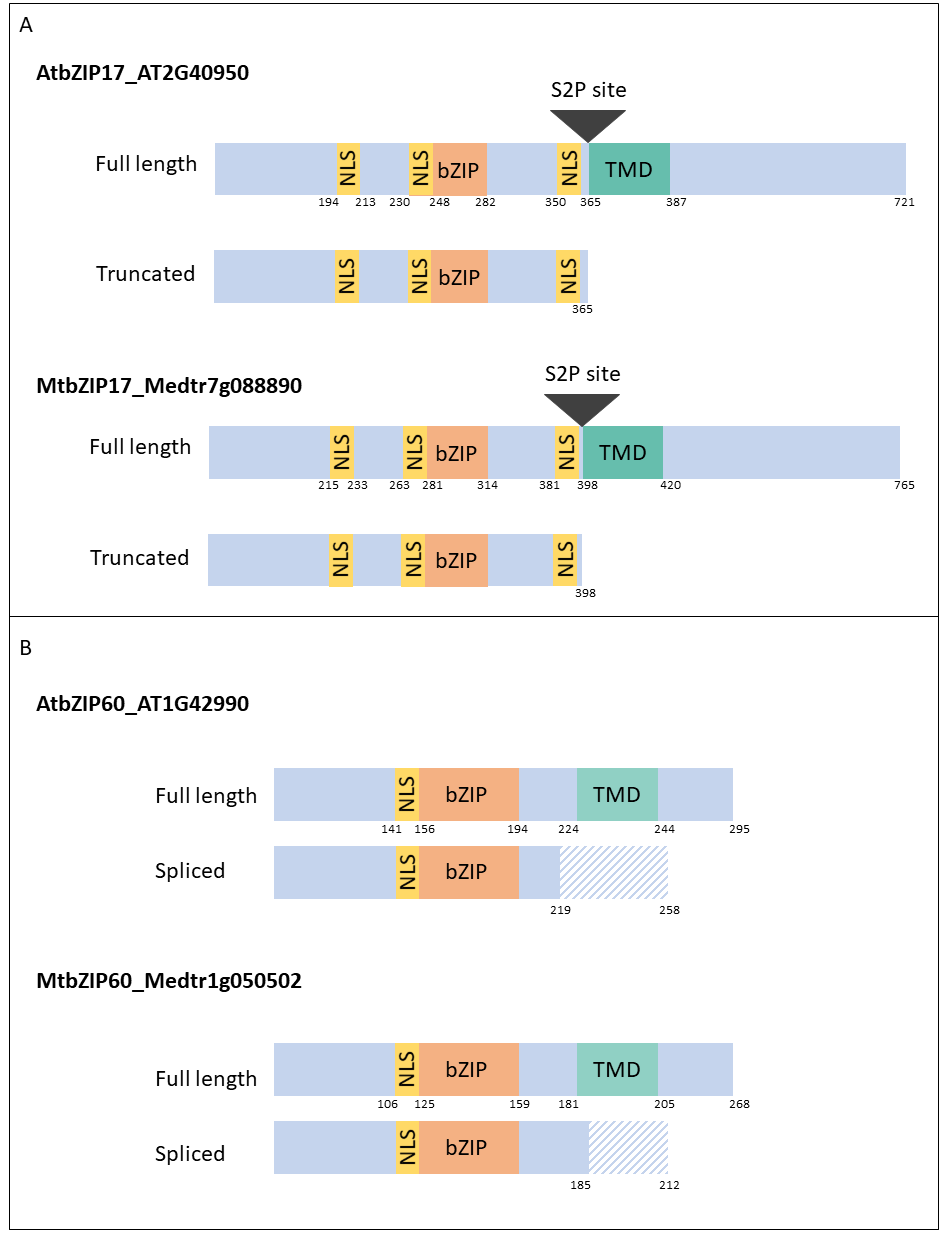
**

**Supplementary Figure 5.** Predicted protein domains of full-length and truncated bZIP17 and bZIP60 in *A. thaliana* and *M. truncatula*. **A,** Schematization of protein forms and domains between *A. thaliana* bZIP17 (AT2G40950) and the *M. truncatula* ortholog (Medtr7g088890). Black triangles highlight the putative position of Site-2 Protease cleavage sites. For both proteins, the full-length and truncated forms are presented. **B,** Schematic comparison of protein forms and domains of *A. thaliana* bZIP60 (AT1G42990) and the *M. truncatula* ortholog (Medtr1g050502). The striped boxes represent altered protein sequences in the splice variants lacking the TMD. For both panels A and B, the NLS was predicted using subcellular localization prediction software (<http://localizer.csiro.au/>). The localization of the bZIP and TMD domains were determined with InterPro (https://www.ebi.ac.uk/interpro/) by comparison with Conserved Domain Database (NCBI) and Phobius (https://www.ebi.ac.uk/Tools/pfa/phobius/), respectively.


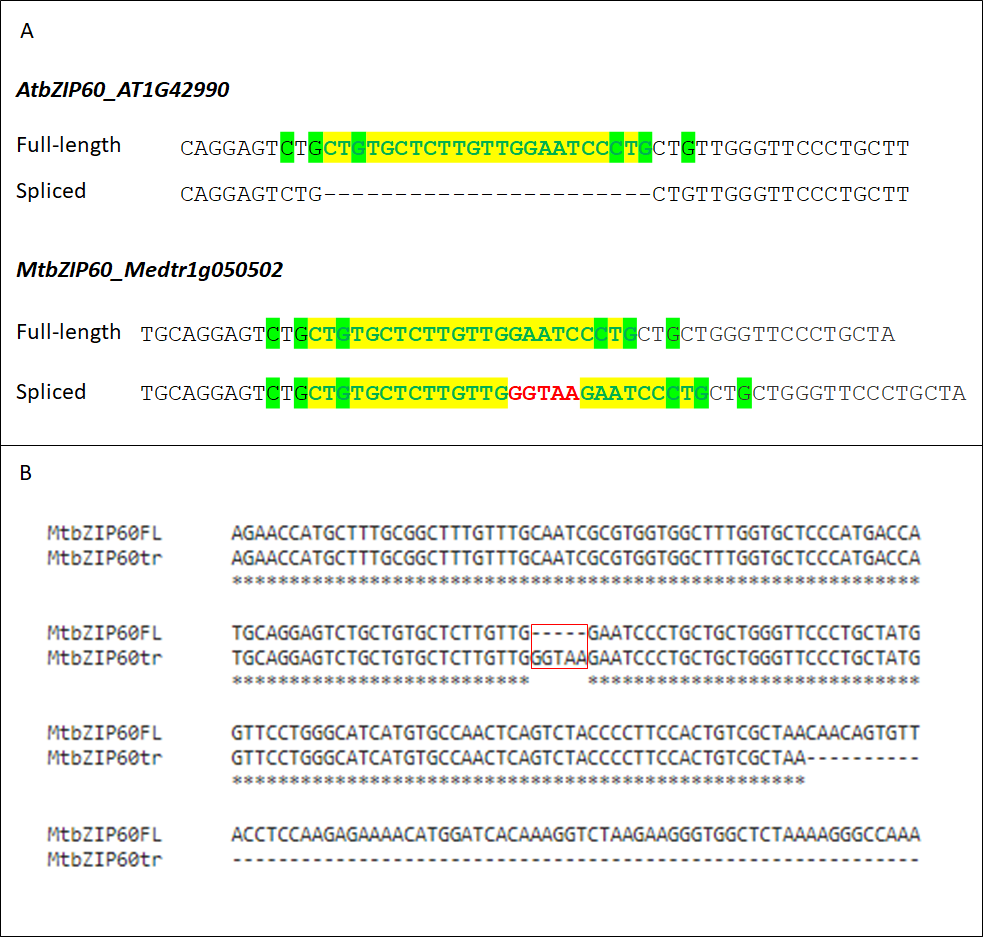


**Supplementary Figure 6.** The unconventional splicing sites of *A. thaliana* and *M. truncatula* *bZIP60* are differently processed. **A,** *bZIP60* unconventional splicing site for *A. thaliana* *AT1G42990* (as described by Nagashima et al., (2011)) and the *M. truncatula* ortholog *Medtr1g05052* (obtained from ensemble database). Green boxes represent signature residues of unconventional splice sites recognized by the IRE1 RNase (Nagashima et al., 2011). Green letters highlighted in yellow are conserved sequences surrounding the splicing sites found in both genes. Differently from *A. thaliana*, in *M. truncatula bZIP60* splicing causes an insertion of a few base pairs (red letters) that leads to a frameshift and translation of a truncated protein form that lacks the transmembrane domain. **B,** ClustalW transcript alignment of *M. truncatula bZIP60* splice variants neighboring the splicing site. Within the red box, the inserted base pairs of the truncated variant (tr) are highlighted.

**
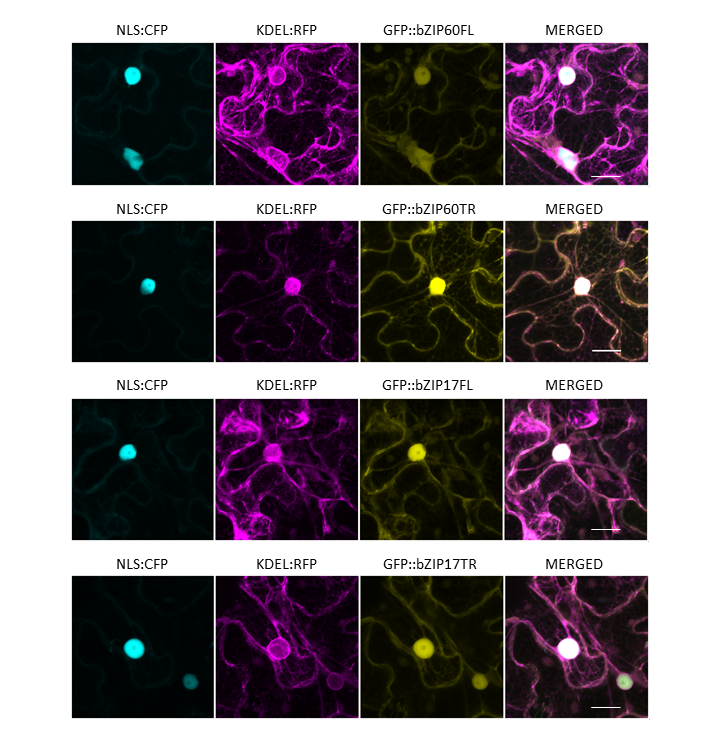
**

**Supplementary Figure 7.** Localization of full-length and truncated bZIP17 and bZIP60. Localization studies on candidate GFP-tagged bZIPs coexpressed with fluorescent markers for the nucleus (NLS:CFP) and endoplasmic reticulum (KDEL:RFP). Confocal images were taken 72 h after *Agrobacterium*-mediated transient expression in the epidermal cells of *N. benthamiana* leaves. bZIP60 and bZIP17 in either their truncated (TR) or full-length (FL) form were predominantly observed in the nucleus as well as in the endoplasmic reticulum (ER). Bars = 20 μm.****

**Supplementary Figure 8.** *M. truncatula* *bZIP17Δ* and *bZIP60Δ* do not affect transactivation of TS biosynthesis gene promoters by themselves. The Y-axis shows fold change in normalized fLUC activity relative to the control transfection with *proCaMV35S:GUS.* The error bars designate SE of the mean (n=8 biological repeats). Different letters indicate statistically significant differences at P < 0.05, as determined by ANOVA, *post hoc* Tukey analysis.

**Supplementary Figure 9.** Analysis of TS gene expression in bZIP17 gain-of-function *M. truncatula* hairy root lines. **A,** Hairy root phenotype of CTR and bZIP17∆-OE hairy roots. **B,** qPCR analysis of *bZIP17∆*, *bZIP17*, *bZIP60*, *BIP1/2* genes in three independent CTR and bZIP17∆-OE lines. **C,** qRT-PCR analysis of TS biosynthetic genes in three independent CTR and bZIP17∆-OE lines. Values in the y-axis represent the expression ratio relative to the normalized transcript levels of CTR lines. The error bars designate SE (n = 3, technical repeats). Statistical significance was determined by a Student’s *t*-test (*, P < 0.05, **, P < 0.01, ***, P < 0.001).

**
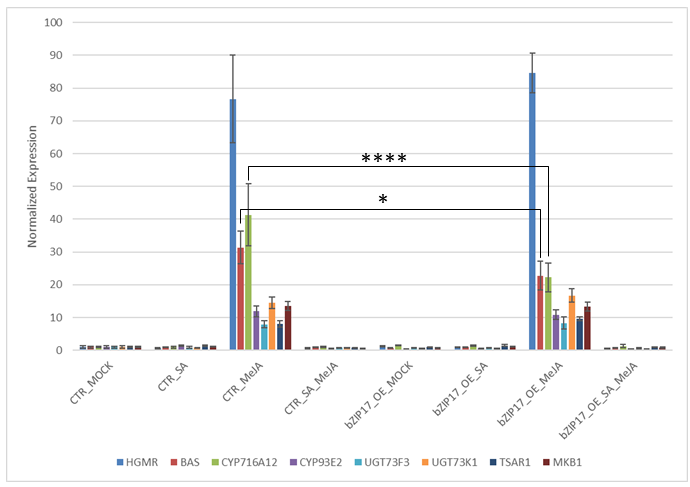
**

**Supplementary Figure 10.** Analysis of TS gene expression in MeJA-treated bZIP17 gain-of-function *M. truncatula* hairy root lines. qRT-PCR analysis of TS biosynthetic genes in three independent CTR and bZIP17∆-OE lines, mock-, MeJA-, or SA-treated for 4 h. Values in the y-axis represent the expression ratio relative to the normalized transcript levels of mock-treated CTR lines. The error bars designate SE (n = 3, corresponding to the independent transformed CTR and bZIP17∆-OE lines generated). Upon MeJA treatment of CTR and bZIP17∆-OE lines, all transcripts were found significantly different (P < 0.0001) to mock-, SA- and SA/MeJA-treated lines. However, MeJA treatment did not result in significant differences in transcript levels between CTR and bZIP17∆-OE lines, except for *BAS*. Statistical significance was determined by a Student’s *t*-test (*, P < 0.05) and *CYP716A12* (****, P < 0.0001).

**Supplementary Figure 11.** Silencing of *bZIP60* slightly increases *CYP716A12* and *UGT73F3* gene expression in *M. truncatula* hairy roots. **A**, Morphology of control (CTR) and bZIP60^KD^ hairy roots. **B-C,** qRT-PCR analysis of *bZIP17*, *bZIP60*, *BIP1/2* genes (**B**) and of TS biosynthetic genes (**C**) in three independent CTR and two independent *bZIP60^KD^* hairy root lines. Values in the y-axis represent the expression ratio relative to the normalized transcript levels of CTR lines. The error bars designate SE (n = 3, technical repeats). Statistical significance was determined by a Student’s *t*-test (*, P < 0.05, **, P < 0.01, ***, P < 0.001).

**Supplementary Figure 12.** *bZIP17* expression is induced under salt stress conditions. Coexpression profiles of *bZIP17* (dark blue, MTR.9115) and *bZIP60* (light blue, Mtr.35426) with *MKB1* (red, Mtr.43815), *TSAR1* (green, Mtr.43316) and *HMGR1* (black, Mtr.10397) in *M. truncatula* under different conditions were generated with the MtGEA tool (He et al., 2009). The blue arrow depicts the peak in *bZIP17* transcripts in NaCl-treated root cultures.


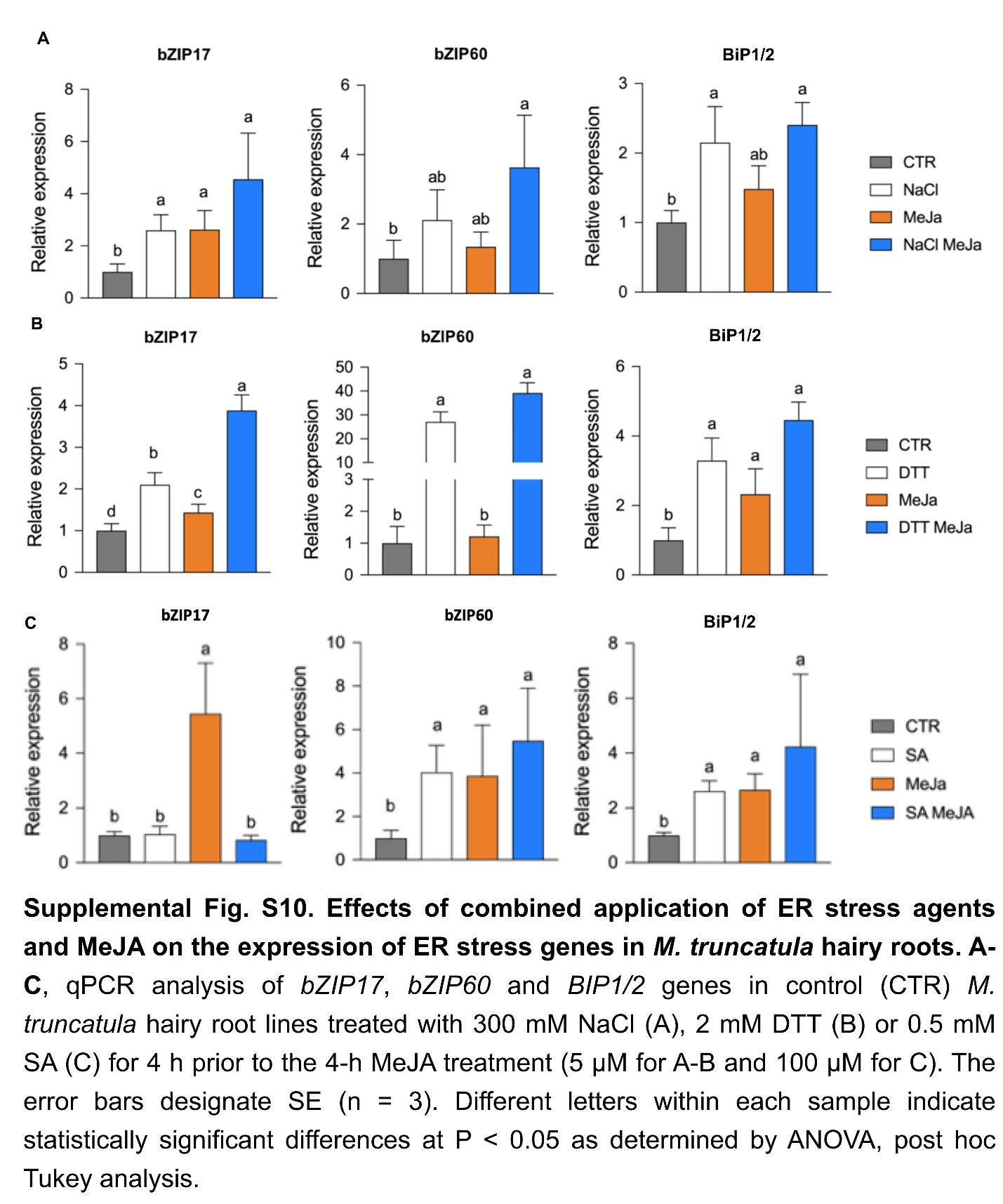


**Supplementary Figure 13.** Effects of combined application of ER stress agents and MeJA on the expression of ER stress genes in *M. truncatula* hairy roots. **A-C,** qPCR analysis of *bZIP17*, *bZIP60* and *BIP1/2* genes in control (CTR) *M. truncatula* hairy root lines treated with 300 mM NaCl (**A**), 2 mM DTT (**B**) or 0.5 mM SA (**C**) for 4 h prior to the 4‑h MeJA treatment (5 µM for A-B and 100 µM for C). The error bars designate SE (n = 3). Different letters indicate statistically significant differences at P < 0.05, as determined by ANOVA, *post hoc* Tukey analysis.

**Supplementary Figure 14.** DTT treatment does not affect MeJA-mediated elicitation of JA pathway genes. qPCR analysis of *JAZ1*, *LOX*, *MYC2a* and *MYC2b* genes in three independent control (CTR) *M. truncatula* hairy root lines. Hairy roots were treated with DTT for 4 h prior to the 4‑h MeJA treatment. The error bars designate SE (n = 3). Different letters indicate statistically significant differences at P < 0.05, as determined by ANOVA, *post hoc* Tukey analysis.


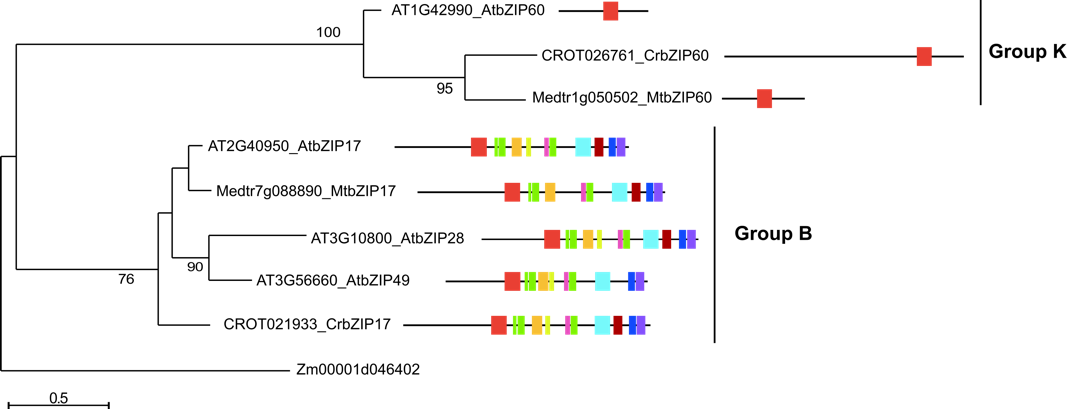


**Supplementary Figure 15.** Phylogenetic analysis of *A. thaliana, M. truncatula* and *C. roseus* bZIP transcription factors of groups B and K. Maximum likelihood phylogenetic tree using the best-fit model JTT+G4 was generated with 1,000 bootstrap replicates using IQTREE. Bootstrap values greater than 70 are given at the nodes. The scale bar indicates number of changes. *Zea mays* bZIP *Zm00001d046402* was included as an out-group. Conserved sequences were determined using MEME (http://meme-suite.org/tools/meme), generating the ten most significantly conserved sequences in the full-length bZIP TFs sequences. The conserved motifs are represented by colored boxes in front of the gene names. The bZIP motif is represented in red. The alignment used to build the phylogenetic tree can be found in Supplementary Figure 16.

**
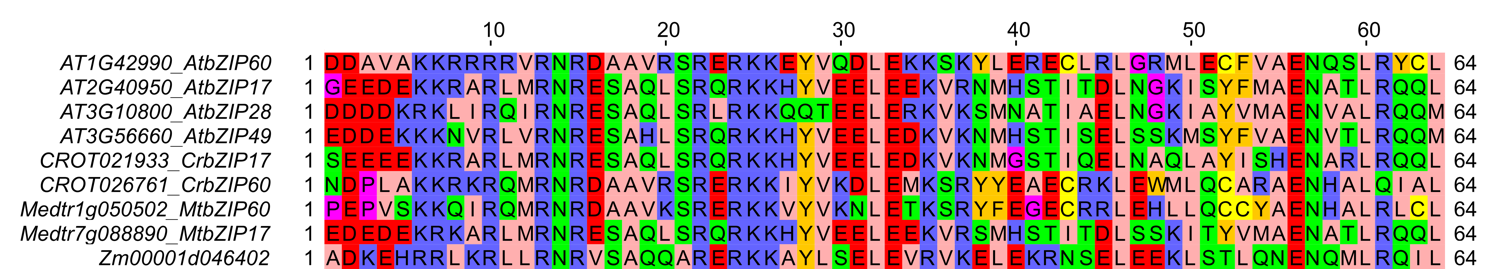
**

**Supplementary Figure 16.** Sequence alignment used for the phylogenetic analysis of *A. thaliana, M. truncatula* and *C. roseus* bZIP transcription factors of groups B and K.
